# A guide for the generation of repositories of clinical samples for research on Chagas disease

**DOI:** 10.1371/journal.pntd.0012166

**Published:** 2024-08-15

**Authors:** Nieves Martínez-Peinado, Juan Carlos Gabaldón-Figueira, Roberto Rodrigues Ferreira, María Carmen Thomas, Manuel Carlos López, Tania Cremonini Araújo-Jorge, Belkisyolé Alarcón de Noya, Soledad Berón, Janine Ramsey, Irene Losada Galván, Alejandro Gabriel Schijman, Adriana González Martínez, Andrés Mariano Ruiz, Gimena Rojas, Roberto Magalhães Saraiva, Oscar Noya-González, Andrea Gómez, Rosa A. Maldonado, Jimmy Pinto, Faustino Torrico, Ivan Scandale, Fernán Agüero, María-Jesús Pinazo, Joaquim Gascón, Alejandro Marcel Hasslocher-Moreno, Julio Alonso-Padilla, On behalf of the NHEPACHA Network (New Tools for the Diagnosis and Evaluation of Chagas Disease)

**Affiliations:** 1 Barcelona Institute for Global Health (ISGlobal), Hospital Clínic - University of Barcelona, Barcelona, Spain; 2 Secció de Parasitologia, Departament de Biologia, Sanitat i Medi Ambient, Facultat de Farmàcia i Ciències de l’Alimentació, Universitat de Barcelona, Barcelona, Spain; 3 Laboratory of Innovations in Therapies, Education and Bioproducts, Oswaldo Cruz Institute, Oswaldo Cruz Foundation (LITEB-IOC/Fiocruz), Rio de Janeiro, Brazil; 4 Laboratory of Applied Genomics and Bioinnovations, Oswaldo Cruz Institute, Oswaldo Cruz Foundation, (LAGABI-IOC/Fiocruz), Rio de Janeiro, Brazil; 5 Instituto de Parasitología y Biomedicina López Neyra, Consejo Superior de Investigaciones Científicas (IPBLN-CSIC), PTS-Granada, Granada, Spain; 6 Instituto de Medicina Tropical, Facultad de Medicina, Universidad Central de Venezuela, Caracas, Venezuela; 7 Fundación Mundo Sano, Buenos Aires, Argentina; 8 Centro Regional de Investigación en Salud Pública (CRISP), Instituto Nacional de Salud Pública (INSP), Tapachula, Chiapas, México; 9 Hospital Universitario 12 de Octubre, Madrid, Spain; 10 Laboratorio de Biología Molecular de la Enfermedad de Chagas, Instituto de Investigaciones en Ingeniería Genética y Biología Molecular “Dr. Héctor N. Torres” - INGEBI-CONICET, Buenos Aires, Argentina; 11 Departamento de Investigación, Salvando Latidos A.C., Guadalajara, Mexico; 12 Departamento de Investigación, Instituto Cardiovascular de Mínima Invasión (ICMI), Guadalajara, Mexico; 13 Instituto Nacional de Parasitología “Dr Mario Fatala Chaben” ANLIS MALBRÁN, Ministerio de Salud, Buenos Aires, Argentina; 14 CONICET, Consejo Nacional de Investigaciones Científicas y Técnicas, Buenos Aires, Argentina; 15 Universidad Mayor de San Simón and Fundación CEADES, Cochabamba, Bolivia; 16 Evandro Chagas National Institute of Infectious Diseases, Oswaldo Cruz Foundation, Rio de Janeiro, Brasil; 17 Cátedra de Parasitología, Escuela ¨Luís Razetti” Facultad de Medicina, Universidad Central de Venezuela, Caracas, Venezuela; 18 Centro para Estudios Sobre Malaria, Instituto de Altos Estudios “Dr. Arnoldo Gabaldón”, Ministerio del Poder Popular para la Salud (MPPS), Caracas, Venezuela; 19 Department of Biological Sciences, The University of Texas at El Paso, El Paso, Texas, United States of America; 20 Drugs for Neglected Diseases Initiative (DND*i*), Geneva, Switzerland; 21 Instituto de Investigaciones Biotecnológicas (IIB)–Consejo Nacional de Investigaciones Científicas y Técnicas (CONICET), San Martín, Buenos Aires, Argentina; 22 Escuela de Bio y Nanotecnologías (EByN), Universidad de San Martín (UNSAM), San Martín, Buenos Aires, Argentina; 23 CIBER de Enfermedades Infecciosas, Instituto de Salud Carlos III (CIBERINFEC, ISCIII), Madrid, Spain; Wadsworth Center, UNITED STATES OF AMERICA

## Abstract

Chagas disease, caused by the parasite *Trypanosoma cruzi*, affects over 6 million people, mainly in Latin America. Two different clinical phases, acute and chronic, are recognised. Currently, 2 anti-parasitic drugs are available to treat the disease (nifurtimox and benznidazole), but diagnostic methods require of a relatively complex infrastructure and trained personnel, limiting its widespread use in endemic areas, and the access of patients to treatment. New diagnostic methods, such as rapid tests (RDTs) to diagnose chronic Chagas disease, or loop-mediated isothermal amplification (LAMP), to detect acute infections, represent valuable alternatives, but the parasite’s remarkable genetic diversity might make its implementation difficult. Furthermore, determining the efficacy of Chagas disease treatment is complicated, given the slow reversion of serological anti-*T*. *cruzi* antibody reactivity, which may even take decades to occur. New biomarkers to evaluate early therapeutic efficacy, as well as diagnostic tests able to detect the wide variety of circulating genotypes, are therefore, urgently required. To carry out studies that address these needs, high-quality and traceable samples from *T*. *cruzi-*infected individuals with different geographical backgrounds, along with associated clinical and epidemiological data, are necessary. This work describes the framework for the creation of such repositories, following standardised and uniform protocols, and considering the ethical, technical, and logistic aspects of the process. The manual can be adapted according to the resources of each laboratory, to guarantee that samples are obtained in a reproducible way, favouring the exchange of data among different work groups, and their generalizable evaluation and analysis. The main objective of this is to accelerate the development of new diagnostic methods and the identification of biomarkers for Chagas disease.

## Author summary

The diagnosis of Chagas disease requires costly equipment and trained personnel, which unavailability hinders access to diagnosis, and treatment, in vast areas of endemic regions. Additionally, timely assessment of treatment efficacy is complicated due to the slow reversion of serological anti-*T*. *cruzi* reactivity. Hence, there is an urgent need for biomarkers of early therapeutic efficacy and disease prognosis, as well as more practical diagnostic tools. To conduct studies that can address these needs, it is essential to have collections of clinical samples with good quality, traceability, and appropriately associated clinical-epidemiological information. In this work, we provide a standard protocol to collect, process, store, and transport clinical samples from Chagas disease patients. The manual was produced upon reaching a consensus among the experts within the NHEPACHA network, a coalition of clinical and academic researchers from the Americas and Spain that pursues the identification and validation of new biomarkers and diagnostics for Chagas disease.

## 1. Introduction

Chagas disease, caused by protozoan parasite *Trypanosoma cruzi* (*T*. *cruzi*), affects over 6 million people worldwide, and it is the most significant parasitic zoonosis in Latin America [1]. Furthermore, due to migratory flows, it has become a global problem during the last decades, with cases being regularly diagnosed in Europe, North America, Oceania, and Asia [2].

The disease presents acute and chronic phases. The first one, lasting 4 to 8 weeks, is mostly asymptomatic but can be lethal in 5% of cases, particularly in children and immune-suppressed patients. Most of the infected individuals progress to a chronic phase [3], which can last for decades along which the parasite presence persists without causing clinical symptoms. Nonetheless, over time, approximately 30% of those chronically infected individuals will develop the characteristic cardiac and/or digestive manifestations of the disease [3].

Since almost 60 years ago, there are 2 anti-parasitic drugs available: benznidazole and nifurtimox. Both are highly efficacious during the acute phase, but less so in the chronic one, when most cases are diagnosed. Apart from this, both require long treatment schemes, frequently associated with adverse effects that can lead to treatment interruption. Early diagnosis is crucial to enhance treatment efficacy, yet symptoms in the acute phase are often nonspecific, hindering its timely detection [1].

The diagnosis of the chronic infection phase is indirect and relies on the detection of specific anti-*T*. *cruzi* immunoglobulins. Given the parasite’s remarkable antigenic diversity, the World Health Organization (WHO) and the Pan-American Health Organization (PAHO) recommend the use of 2 serologic tests detecting different antigenic sets in order to reach a confirmed diagnosis of the infection. Serological techniques present certain inconveniences, such as the possible cross-reactivity with immunoglobulins against other closely related parasites, like *Leishmania* spp., or the impossibility to assess the efficacy of treatment due to the long period needed for antibody titres to return to normal following successful treatment [4]. This is also problematic in the context of clinical trials evaluating new drugs against the disease. Also, serological techniques require trained personnel and relatively expensive equipment to be performed, making their regular use in most primary care facilities of endemic regions unfeasible. In response to this problem, inexpensive immunochromatographic methods, known as rapid diagnostic tests (RDTs) were developed recently [5,6]. These are easy to use, require no electricity or temperature control, and can provide a result in less than an hour.

Although RDTs are used for screening in countries like Bolivia, Colombia, or Paraguay, conventional serological methods remain necessary to confirm a diagnosis [6]. An alternative that has been proposed is the combined use of 2 (or 3 in case of discordancy) RDTs with different antigenic targets [7–11]. While RDTs have shown high sensitivity and specificity in some regions with a high infection prevalence (like certain areas of Bolivia, northern Argentina, and in Colombia), their performance is questioned across other geographic regions, where different circulating parasite strains may predominate, as well as in areas of low infection prevalence [6,12]. Hence, the development of a “universal” RDT, or regionally tailored RDTs with an ample geographic coverage, represents a very attractive objective.

On the other hand, the lack of biomarkers for the evaluation of disease prognosis and the assessment of treatment efficacy in Chagas disease pose a significant challenge for patients, medical providers, and clinical researchers. Such biomarkers could identify those at higher risk of organ damage and facilitate the development of new tests to assess the therapeutic efficacy of currently available drugs or those under clinical trial [12–14].

Identifying novel response to treatment and progression biomarkers would mean a major breakthrough in the field. Diverse candidate molecules have been evaluated, but most studies have only included a limited number of clinical samples from patients with short posttreatment follow-up periods [12]. As for diagnostic tests, larger, thoroughly classified and characterised cohorts of diverse geographic origin are required to address this problem. These cohorts should encompass uninfected controls and individuals with seroconversion after prolonged monitoring periods to confidently identify positive cure controls. Evaluating early therapeutic response markers requires extended participant follow-up, ideally spanning 5 years or more posttreatment, to study the chronic progression of the disease [13]. For the analysis of the samples following similar methodologies to be comparable across different regions/countries, those should always be obtained, processed, and stored following standardised, reproducible protocols, and be linked to clinic-epidemiological data of quality.

Biological samples can be stored in biobanks and/or research biorepositories. While there is no universally accepted concept for a biobank, this can be defined as a non-for-profit public or private facility, where biological samples are stored and annotated with relevant metadata. These samples can be used by third-parties in research or healthcare projects. Biobanks are typically understood as centralised spaces that receive samples from different biorepositories. Unlike biobanks, biorepositories typically belong to individual research groups and are not regularly shared with other groups.

In this manual, we describe the features to consider when generating and maintaining a collection of clinical samples obtained from patients with Chagas disease. Detailed information was prepared in accordance with the BRISQ (Biospecimen Reporting from Improved Study Quality, [15]). The described methodology is the result of a collaboration within the research network NHEPACHA (acronym for New Tools for the Diagnosis and Evaluation of patients with Chagas disease initiative, reading in Spanish).

NHEPACHA was created in 2011, with the objective of identifying and validating the use of novel biomarkers against Chagas disease. It is currently formed by 18 research groups from 9 countries. The framework described in this paper was reviewed and approved by the network’s expert commission. Its final goal is to facilitate the creation of new collections of clinical samples obtained from patients with Chagas disease, as well as to improve existing ones, so they can be used in international, multi-centre studies for the evaluation of new therapeutic alternatives, and the identification of biomarkers of therapeutic efficacy and patient progression. Moreover, this work has been translated to Spanish and Portuguese in order to make it universally accessible and available (S3–S8 Files).

## 2. Ethics

The extraction, processing, and usage of samples must be carried out in total accordance with the current revision of the Helsinki Declaration (64th Assembly General, Fortaleza, Brazil, 2013), as well as in agreement with the respective local regulation of the country where the samples are collected. Furthermore, adhering to European legislation, known for its stringent data protection measures, could streamline the transfer of samples for future research projects.

Samples should only be collected after the participant has signed an informed consent form (IC). The IC must include the objective/s of the research and the intended use of the samples (present and future, if needed). Both, the IC, as well as the corresponding study protocol must have been approved by an independent clinical research ethical committee. This committee will confirm that the project complies with existing legal and ethical regulations, while researchers are responsible of the traceability of samples and the confidentiality of any derived data.

The biological material, as well as the clinical data associated to it, must be included in the collection, and made available to any research group that may require them, after the corresponding ethical approval has been obtained. Biological samples and their associated metadata might be stored as long as the period of time described in the specific study protocol and IC forms. Both the samples and their metadata should always be coded or anonymized, and only authorised personnel should be able to link such codes with a participant’s identity. Ideally, clinical data and any other relevant information should be stored in both physical and electronic records, managed by the responsible study centre. This information can only be handled and shared in the context of an ongoing biomedical research project and in accordance with its approved protocols and IC forms.

The retrospective use of samples for purposes not disclosed at the time of collection should be in accordance with the information included in the IC forms. If not, another IC signed by the participant should be obtained. On the other hand, requesting biological samples that have already been collected, and their metadata, poses various challenges that should be considered: stringent procedures and guidelines, encompassing permissions, documentation, and compliance with ethical and regulatory standards that may change between countries.

## 3. Sample extraction

The type of samples extracted and their processing (Section 4: Sample processing) will be determined based on the nature of the assays in which they will be used. If possible, participants should be recruited as soon as their *T*. *cruzi* infection is confirmed, trying to guarantee their timely assistance to follow-up visits. Depending on the type of study planned, participants who have started anti-parasitic treatment might have to be excluded, regardless of whether this was completed or not. This would be the case, for instance, in studies evaluating the performance of new serological diagnostics (e.g., RDTs). The infection status of an individual is unlikely to be known during the first visit. Even after the first samples are collected, the diagnosis can take up to several weeks, particularly in endemic areas. Besides, completing with the clinical data form will require of a series of exams (e.g., electrocardiography), and that information will have to be linked to the collected sample.

The extraction of the samples should be made in a healthcare centre with the appropriate infrastructure. The time between sample collection and the next follow-up visits will depend on the diagnosis obtained during the first visit. Normally, and according with current clinical recommendations, samples from people with the infection that comply with criteria to receive anti-parasitic treatment should be obtained immediately before and after completing the treatment scheme, as well as 6 months after finishing it, and once a year from that point onwards. Samples from uninfected controls should ideally be obtained 1, 5, and 10 years after the study recruitment visit. In all cases, samples must be correctly labelled with the corresponding coding for each patient, the extraction date, and the type of sample obtained. The sample coding will be paired with that of the laboratory responsible for processing. Certain flexibility of the visits window (e.g., 2 to 3 months earlier or later from the scheduled date) should be allowed due to the challenges of keeping up to the agenda.

**Fig 1:**
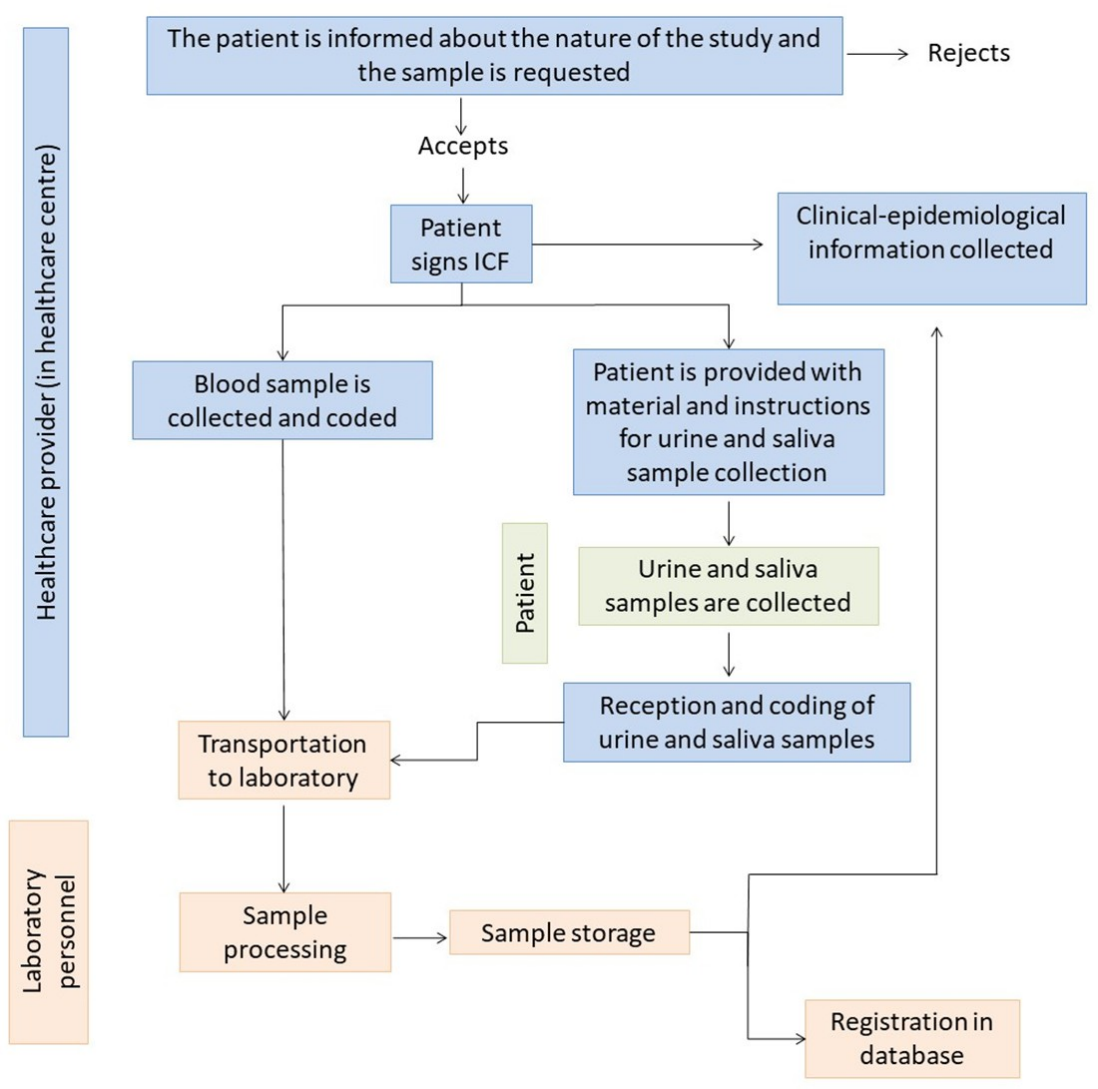
Sample collection, transportation, storage, and processing algorithm.

Once collected, samples should be ideally transported to the reference laboratory at 4°C and stored at that temperature until processing, which should be done within a period no longer than 24 h after the extraction (Section 4: Sample processing). Fig 1 represents the sample collection, transportation, storage, and processing algorithm.

### 3.1. Blood extraction

#### 3.1.1. General procedures for blood extraction

Blood extraction must be performed by trained personnel to reduce the participant’s discomfort, without compromising the sample’s quantity and quality.

It is usually obtained by venous puncture using either needle and syringe or a plastic exchangeable vacuum extraction system [16]. Venous blood extractions can be made following any technique regularly applied in the health centre. However, certain general recommendations should be followed to reduce the impact of extraction on subsequent analytical processes. For example, the subject should be allowed to sit or lay down, the puncture area disinfected with 70% isopropyl alcohol, used a tourniquet and press the puncture wound at least 2 min to favour haemostasis (S1 File).

**Fig 2:**
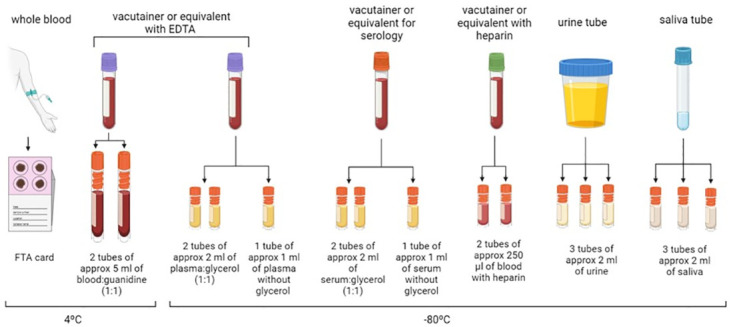
Summary of the samples routinely collected in a Chagas disease clinical laboratory. The suggested number of aliquots and volumes to be collected are shown. (Figure created with Biorender; https://biorender.com/).

For the construction of a samples library, it will be necessary to extract at least 2 samples: one in an anticoagulant-treated tube and another in an untreated tube (Fig 2). For the sake of results’ generalizability and based on the availability of tubes with anticoagulant, ethylenediaminetetraacetic acid (EDTA, usually EDTA-K2 for being the most common) should be preferentially used as the anticoagulant of choice if possible.

#### 3.1.2. Whole blood and its fractions

Three different samples are obtained from a blood extraction: serum, plasma, and whole blood. These samples should be collected in individual tubes and extracted in a sequential order, starting with the untreated tube destined to serum, and followed by EDTA-treated tubes for plasma and whole blood. This is important to avoid cross-contamination of samples with EDTA and similar additives.

i) **Serum** is the most used sample in studies looking for new biomarkers, and for the evaluation of new diagnostic techniques. It must be collected in untreated (dry) tubes. If possible, tubes containing an inert gel to favour serum separation should be used, as they prevent the contamination of the sera with other blood components.
ii) **Whole blood** is useful for the evaluation of RDTs, as well as for performing parasitological techniques, such as the micro-method [4]. It is also the sample most commonly used in the molecular diagnosis of infection, whether by polymerase chain reaction (qualitative and quantitative PCR) or LAMP [17,18]. This sample is typically collected in EDTA-treated tubes, which should be ideally conserved at −80 or −20°C. If samples need to be kept at 4°C for extended periods of time prior to molecular diagnosis, they should be treated with guanidine hydrochloride 6 M–EDTA 0.2 M (GE), pH 8.00 at a 1:1 proportion (vol.:vol). GE is excellent to preserve samples for molecular diagnosis, even if they are stored at room temperature, something particularly useful in endemic areas. Tubes preloaded with GE can also be used to process several samples at the same time. However, guanidine is a costly reagent, which may limit its widespread use in endemic settings. A protocol for the preparation of this reagent is detailed in S2 File.
  Note that if samples are to for the evaluation of LAMP molecular tests based on calcein substrate reactivity (e.g., the *T*. *cruzi-*LAMP developed by Eiken Chemical Co., Tokyo, Japan), it is advisable to avoid the use of EDTA, as it might affect the performance of the assay [18]. Using heparin-treated tubes instead, represents an alternative in these cases. This is too the preferred anticoagulant to use in samples that will be analysed by the direct parasitological microscopy-based micromethod test. While LAMP and PCR have an almost identical sensitivity and specificity [19], the former has the advantage of requiring a simpler infrastructure, facilitating its use in endemic areas, particularly for the diagnosis of congenital Chagas disease.
iii) **Plasma** is obtained from samples collected in anticoagulant-treated tubes, preferably with EDTA, as this is the compound most usually used for the collection of whole blood samples, facilitating the logistical aspects of the sample collection process. Other anticoagulants with different mechanisms of action might compromise sample quality.

If necessary, it is recommended to prioritise the obtention of serum over plasma, as it is easier to obtain and performs better in different diagnostic systems. Nonetheless, it is important to consider that coagulation factors are not present in serum, and these might be useful for the identification of biomarkers involved in the activation of the coagulation pathway [20]. Whole blood sample should be obtained whenever possible, for molecular diagnosis.

#### 3.1.3. Volume of blood extracted

The total volume of blood extracted will depend on the participant’s age. Ideally, the total extracted volume in participants aged 18 years or older should not exceed 10 ml in EDTA-treated tubes (5 ml in each of the 2 tubes desirable) and 5 ml in the untreated tube ➔15 ml of total blood extracted. For participants between 5 and 18 years old, the total extracted volume in EDTA-treated tubes should be 5 ml (2.5 ml in each tube) and 5 ml in the untreated one for the serum ➔ 10 ml of total blood extracted. In children aged 2 to 5 years old, the minimum possible volume of blood should be extracted, in accordance with local paediatric guidelines [21,22]. Neonates and children under 2 years of age constitute another group of interest, primarily for the serological study within congenital Chagas disease diagnosis algorithm. Blood from neonates can be obtained through venous extraction from the back of the hand at birth or through collection of blood drops from the heel (around 500 μl). From 9 months old onwards, it would be possible to obtain around 1 ml of blood by arm venous puncture.

If possible, a fraction of the blood collected (250 to 500 μl) should be transferred to heparin-treated tubes to be able to perform LAMP assays. These assays can also be performed using dried blood spots (DBSs) collected directly on filter paper, e.g., FTA cards (QIAcard Flinders Technology Associates (FTA Classic cards, Qiagen, United Kingdom)) [23].

### 3.2. Collection of urine and saliva

The use of serologic techniques for the diagnosis of *T*. *cruzi* infection in samples of urine [24] or saliva [25] represents a promising noninvasive alternative to existing methods. Therefore, the inclusion of these samples in clinical collections could be considered. However, the utility of urine and saliva is relatively restricted compared to blood when prioritisation is necessary, specifically when considering the space available and costs associated to maintain the samples storage. Urine collection is performed directly by the participant himself/herself, after clear verbal and written instructions have been provided (S1 File). While this sample can be collected at home, it is advisable to do it in the healthcare centre where the other samples are being extracted and during the same visit. Ideally, 5 to 10 ml of the first urine in the morning should be collected [26].

Collection of saliva samples is also performed directly by the participant (S1 File), preferentially at the healthcare centre, same as for the rest of the samples. The participant is required to refrain from drinking anything other than water, eating, smoking, brushing his/her teeth, or chewing gum for at least 1 h before collecting the sample. Ideally, 5 to 10 ml of saliva should be collected.

## 4. Sample processing

All human samples should be considered as potentially dangerous due to their intrinsic biological risk, and therefore must be processed in a type II (BSL-2) laminar flow hood. This will both protect the operator and minimise the risk of contamination of the samples. All tubes should be clearly labelled before processing the samples, indicating the participant’s identification code, the type of sample, and the date of processing.

While it will require more storage space, it is advisable to prepare 2 to 3 aliquots of all the samples processed. For example, ideally 3 aliquots stored in screw cap, O-ring cryotubes should be produced for long-term storage. The production of only 2 aliquots is acceptable, if the total sample volume is limited. In case of limited access to storage facilities, it is possible to prepare a single aliquot and furtherly sub-aliquot such sample after being thawed for the first time. The number of times that a sample has been thawed should be noted, as subsequent freezing/thawing cycles might compromise the sample’s quality. If the sample was prepared by diluting it in an equal volume of glycerol (previously autoclaved and at least 99% pure), it can be thawed up to 10 times. Samples without glycerol should not be thawed more than 3 times. A standard operating procedure for processing samples is presented in S2 File.

### 4.1. Serum

For serum separation, the untreated tube should be centrifuged at 1,600 g for 10 min at room temperature. In the absence of a centrifuge, it is possible to let a clot form and precipitate spontaneously, although this should be avoided when possible. It is recommended to pipet 2 to 3 ml of the sample and mix it with an equal volume of glycerol, before aliquoting the resulting volume in screw cap cryotubes. This process is particularly necessary in places where access to electricity is irregular, as this might compromise the integrity of stored samples. The use of glycerol would prevent the use of the samples for the evaluation of RDT performance or cytokine-production studies. If these studies are to be performed, extra aliquots without glycerol should be prepared.

### 4.2. Whole blood with guanidine

For whole blood processing, the total volume (around 5 ml) of one of the EDTA-treated tubes should be mixed with an equal volume of GE (e.g., ref G3272 at Sigma-Aldrich). This should be usually done in a 15 ml Falcon tube, and the total volume obtained is usually around 10 ml. After vigorously mixing the solution by inversion, two 5 ml parts should be produced and stored in screw cap, O-ring cryotubes to prevent spills. Guanidine is a chaotropic agent that lyses erythrocytes, while preserving the integrity of nucleic acids.

### 4.3. Plasma

For the obtention of plasma, the other EDTA-treated tube should be centrifuged at 1,200 g for 10 min at room temperature. A volume of at least 2 ml should be obtained after centrifuging. As for serum, the resulting plasma volume can be mixed with an equal quantity of glycerol. As previously discussed, the production of extra glycerol-free aliquots might be necessary depending on the objective with which samples were collected. In both cases, it is recommended to prepare aliquots of 1 to 2 ml.

### 4.4. Saliva

Saliva samples should be centrifuged at 1,000 g for 5 min at room temperature to precipitate the mucus. The remaining supernatant should be collected in screw tap cryotubes (1 to 2 ml per tube).

### 4.5. Urine

Urine does not need to be centrifuged. However, it is important to note any alterations observed in the sample (i.e., increased turbidity or the presence of blood), as these might affect the interpretation of results. These samples should also be stored in 1 to 2 ml screw tap cryotubes.

## 5. Storage of samples

Once processed and correctly labelled (a labelling sheet is provided in S2 File), it is necessary to register the location and storage conditions of each sample. This registry should also include the number of times that a given sample has been thawed, along with the corresponding dates and purpose. The number of thawing cycles should also be noted directly on the tube. Table 1 summarises the ideal and acceptable conditions at which different samples can be stored. Most samples can be safely stored at −70°C without compromising their quality, and temperature might in fact contribute to the laboratory’s energy-saving protocols. For samples meant to be used in cytokine production studies, they must always be stored at −70°C or below.

**Table 1:** Recommended temperatures for the long-term storage of clinical samples.

Sample	Ideal temperature (°C)	Acceptable temperature (°C)
**Whole blood-guanidine or FTA cards**	4	Room temperature
**Whole blood-EDTA/heparin**	−80	−20
**Serum**	−80	−20
**Plasma**	−80	−20
**Saliva**	−80	−20
**Urine**	−80	−20

In endemic regions, where the storing of samples at −80°C is logistically cumbersome, or where access to electricity is irregular, serum and plasma samples can be mixed with sodium azide and stored at 4°C for long periods of time without compromising their quality. However, this is only acceptable for samples to be used in serologic diagnostic tests and can also be done to reduce the number of thawing cycles to which controls used in these tests are exposed to. However, this method should not be used to store samples intended for biomarker-identification studies.

## 6. Data management and annotation with clinical and laboratory information

In order to be actually useful in research, any collection of clinical samples must be properly annotated with their associated clinic-epidemiological data. Similarly, the IC forms signed to obtain the samples should also be stored. It is essential to immediately register any changes in the collection (including the total or partial use, and disposal of samples). In light of these related but distinct entries of information, it is highly advisable to keep all records in a centralised dataset, whose level of sophistication will depend of each laboratory’s available resources and the number of samples handled, ranging from paper-based records to real-time, multiuser informatic datasets.

These datasets must include the exact location of each sample and any relevant clinical and epidemiological information. To facilitate the data collection process, it is very important to prepare questionnaires for the capture, validation, storage, and management of the resulting metadata. While these questionnaires can be paper based, it will be highly recommendable to store a digital copy of each one of them. In these questionnaires (such as the one elaborated by González and colleagues in the NHEPACHA network, and currently submitted for review), clinical and epidemiological information is recorded, as well as laboratory information corresponding to each type of sample, volume, and date of collection and processing, along with any possible incident related to it.

## 7. Sample transportation

Before any samples can be transferred from one centre to another, an agreement should have been signed between both institutions, and any legally mandated requirements must be fulfilled. Typically, a material transfer agreement (MTA) between the 2 centres must be arranged in advance, and a list of the transported samples along with any required importation permits and a customs invoice are also necessary beforehand.

Once all the required documentation is completed, samples should be packaged in compliance with existing legislation, and according to their biological risk classification, which must be declared by the shipping institution, following the guidelines from the corresponding transportation authority.

To ensure the quality of shipped samples, transportation temperature must be monitored and maintained. For this, it is advisable to include a temperature data-logger in the package if possible. Ideally, serum, plasma, urine, and saliva samples should be transported frozen in dry ice at a constant temperature of −70°C. Note that dry ice is classified as a dangerous material and must be appropriately labelled [26]. If it is not possible to guarantee this temperature, samples can be transported at −20°C or using cooling gel packages. In some cases, transportation at room temperature may be acceptable, particularly if samples have been mixed with glycerol, though it is important to note that they would have been thawed once before shipment.

In all cases, a package large enough to contain the samples and the chosen refrigeration method must be used. It is advisable to place the samples between 2 layers of the cooling agent to guarantee the stability of their temperature. The receiving institution is responsible for verifying the content of the package upon arrival and communicate any unexpected occurrence to the sender as soon as possible.

## 8. Conclusion

The procedures described in this manual are meant to guide the standardised establishment of new and improved repositories of clinical samples for the study of Chagas disease. High-quality, traceable samples are essential for the identification of new biomarkers for the diagnosis, and prognosis of Chagas disease, and to monitor the response of patients to treatment. It is recommended to use this guide in combination with the clinical questionnaire guide (by Gonzalez and colleagues) for each sample.

## Supporting information

S1 File Standard operating procedure (SOP) for the collection of clinical samples for their use in Chagas disease research repositories. (DOCX)

S2 File Standard operating procedure (SOP) for the processing of clinical samples for their use in Chagas disease research repositories. (DOCX)

S3 File A guide for the generation of repositories of clinical samples for research on Chagas disease (Spanish). (DOCX)

S4 File Standard operating procedure (SOP) for the collection of clinical samples for their use in Chagas disease research repositories (Spanish). (DOCX)

S5 File Standard operating procedure (SOP) for the processing of clinical samples for their use in Chagas disease research repositories (Spanish). (DOCX)

S6 File A guide for the generation of repositories of clinical samples for research on Chagas disease (Portuguese). (DOCX)

S7 File Standard operating procedure (SOP) for the collection of clinical samples for their use in Chagas disease research repositories (Portuguese). (DOCX)

S8 File Standard operating procedure (SOP) for the processing of clinical samples for their use in Chagas disease research repositories (Portuguese). (DOCX)

S1 Acknowledgements Membership to the NHEPACHA network. (DOCX)
