# Supplementary material for: A guide for the generation of repositories of clinical samples for research on Chagas disease
Source: PLoS Negl Trop Dis. 2024 Aug 15;18(8):e0012166. doi: 10.1371/journal.pntd.0012166 (PMC11326570; doi:10.1371/journal.pntd.0012166)
Supplement: S1 File — (DOCX) [file pntd.0012166.s001.docx]

**S1 POE. Standard operating procedure for the collection of clinical samples for their use in Chagas’ disease research repositories**

1. **Blood extraction**

**Required material:**

- Tray
- Cotton
- Isopropyl alcohol
- Tourniquets
- Surgical gloves
- Vacutainer needle and tubes
- Tube rack
- 1 Untreated collection tube.
- 2 EDTA-K2-treated collection tubes.
- Hypoallergenic surgical tape
- Permanent marker
- Adhesive identification labels
- Biohazard disposal container

**Procedure for the collection of venous blood samples**

1. Verify the patient’s identity and test orders. Remember to collect the sample in the untreated tube first, to prevent contamination with additives.
2. Clearly explain the procedure to the patient.
3. Prepare the extraction material: tourniquet, cotton, alcohol, tape, vacutainer needle and tubes, and gloves should all be on the tray.
4. Label each tube with the corresponding patient code, the date and the type of sample being collected.
5. Prepare the vacutainer needle and apply the tourniquet with sufficient pressure to help you locate the vein, without compromising the blood flow. Excessive pressure might cause haemolysis, venous collapse, and pain, while also compromising the sample’s quality.
6. Select the most prominent and easily palpable vein on the antecubital region, rub alcohol to disinfect the area and let it dry. Puncture the skin with the needle’s bezel facing up and forming an angle of approximately 15º with the surface.
7. If samples are being collected on FTA cards, apply drops of blood directly on them. Fill each circle printed on the FTA card with one drop of blood. It is important that each circle has approximately the same volume of blood, and that the printed area is completely filled.
8. Store the FTA card in a metal bag with silica gel and keep it at 4ºC until transported to a laboratory.
9. Press the cap of the vacuum tube against the free end of the vacutainer needle and let it fill. Once filled, remove the tube from the vacutainer needle and repeat the procedure with the remaining tubes. Remember to collect the sample in the untreated tube first.
10. Remove the last tube from the vacutainer needle, retire the tourniquet and the needle from the patient and press the puncture site with a cotton.
11. Discard the vacutainer needle in the corresponding biohazard container.
12. EDTA-K2-treated tubes must be filled until the vacuum disappears to maintain an adequate proportion between the sample and the anticoagulant. Once filled, immediately mix by inversion 5-10 times to prevent any clots from forming.
13. Confirm that all required samples have been collected before allowing the patient to leave.
14. Store all the collected samples at 4ºC until transported to the laboratory. Samples should be processed within 24 hours after being extracted.
15. **Saliva collection**

**Required material:**

- Saliva collection tube.

**Procedure for the collection of saliva samples**

The patient must be provided the following instructions verbally and in written.

1. Do not eat, chew gum, smoke, or drink anything other than water during at least one hour before collecting the sample. The use of of lipstick, lip balms or creams, and steroid inhalers, should also be avoided immediately before collecting the sample. Avoid any activities that might cause gingival bleeding, including brushing your teeth. Do not use ascorbic acid (vitamin C), or any mouthwash to stimulate salivation before collecting the sample.
2. Rub your palate and cheeks with your tongue to stimulate salivation.
3. Spit into the collection tube until the level of saliva (not including bubbles) reaches the 5 ml line. Do not collect more than 10 ml. Gently tap the tube against a hard surface to reduce the volume of bubbles.
4. Tightly close the collection tube.
5. **Urine collection**

**Required material:**

- Urine container.

**Procedure to collect urine samples**

The patient must be provided the following instructions verbally and in written.

- Wash your hands with water and soap.
- Separate the vaginal labia with your hand, or retract the penis’ foreskin and start urinating. Do not collect the first stream of urine.
- Continue urinating into the sterile collection container, avoiding any contact between your skin and the stream.
- Tightly close the collection container without touching its interior.
