# Supplementary material for: A guide for the generation of repositories of clinical samples for research on Chagas disease
Source: PLoS Negl Trop Dis. 2024 Aug 15;18(8):e0012166. doi: 10.1371/journal.pntd.0012166 (PMC11326570; doi:10.1371/journal.pntd.0012166)
Supplement: S2 File — (DOCX) [file pntd.0012166.s002.docx]

**S2 POE. Standard operating procedure for the processing of clinical samples for their use in Chagas’ disease research repositories**

**Sample collection summary**

|  | **Total blood (approx. 15 ml)** |
| --- | --- |
| **Conditions** | - 10 ml collected in 2 EDTA-treated tubes (violet cap). - 5 ml collected in one untreated tube, with pro-coagulant gel (red cap). - 500 µl collected in a heparin-treated tube (green cap). |
| **Storage of samples until collection by laboratory personnel** | 4ºC |
| **Transportation** | Appoint a person to collect and transport the samples (samples must be processed within the first 24 hours following extraction). |

**General notes**

- All samples must be processed in a BSL-2 hood.
- Individual adherent labels must be provided with the samples prior to processing. Once applied to each tube, labels should be covered with transparent plastic tape, to prevent their degradation.
- After processing the samples, the repository dataset should be updated immediately.

**Reagents**

- Guanidine (6M) EDTA (0.2 M) mixture (pH 8.00): The mixture can be prepared by mixing guanidine hydrochloride, molecular biology grade ≥99% (Ref: G3272, Sigma-Aldrich, MW: 95.53 g/mol) with 174.5 ml molecular biology grade H_2_O (Ref. W4502, Sigma-Aldrich), and filtering through a 0.22 µm filter.
- Glycerol (99% purity, Ref: G5516, Sigma-Aldrich): Autoclaved.
- Mili-Q H_2_O: Autoclaved.

**Materials:**

- 5 ml serological pipettes.
- 15 ml Falcon tubes.
- 2 ml screw cap cryotubes.
- 5 ml screw cap cryotubes.
- 4 ml EDTA-K2-treated tube (violet cap) x 2
- 10 ml untreated tube with pro-coagulant gel (red cap).

**Protocol:**

1. Label all the tubes to be used for processing the samples and cover each label with transparent plastic tape.

Note 1: It is convenient to label all the tubes before starting processing the samples, to avoid mixing up tubes if processing several samples at the same time.

Note 2: The volumes of samples described in this protocol correspond to those obtained from patients 18 years old and older. While volumes used when processing samples from participants under this age are different, the processing procedure is the same.

- **Whole blood (10 ml): Collected in two individual vacutainer, violet capped tubes. Two different samples are obtained from these tubes:**
  - Whole blood + guanidine (tube 1):

1. Transfer the total volume of blood in one of the tubes to a 15 ml Falcon tube.
2. Add the same volume of guanidine hydrochloride (6M) – EDTA (0.2M).
3. Mix by inversion and divide the resulting volume in two 5 ml screw cap cryotubes.
   - Plasma (tube 2):
4. Centrifuge the remaining EDTA-treated tube with whole blood at 1200 g for 10 minutes at room temperature.
5. Transfer the maximum possible volume of plasma to a 15 ml, without disturbing the pellet.
6. Transfer approximately 1/3 of the volume of plasma and prepare 2 ml aliquots in screw cap cryotubes.
7. Add a volume of glycerol equal to the remaining plasma volume in the 15 ml tube.
8. Mix by pipetting until the solution looks homogenous.
9. Aliquot the remaining volume in 2 ml screw cap cryotubes.
10. Note the number of resulting aliquots, as well as their volume in the dataset.
11. Store the aliquots at -80 ºC.

- **Whole blood in untreated tube for serum separation (tube 3, red cap)**

1. Centrifuge the tube at 1600g for 10 min at room temperature.

Note: This tube can be left to clot without centrifuging, but this should be avoided if possible.

1. Transfer the maximum possible volume of serum to a 15 ml Falcon tube.
2. Transfer 1/3 of the collected volume and prepare aliquots in 2 ml screw cap cryotubes.
3. Add a volume of glycerol equal to the remaining plasma volume in the 15 ml tube.
4. Mix by pipetting until the solution looks homogenous.
5. Aliquot the remaining volume in 2 ml screw cap cryotubes.
6. Note the number of resulting aliquots, as well as their volume in the dataset-
7. Store the aliquots at -80 ºC.

- **Whole blood in heparin-treated tube (or equivalent) for LAMP assays (tube 4)**

1. Prepare 2 aliquots of 250 µl.
2. Note the number of resulting aliquots, as well as their volume in the dataset, and store the aliquots at -80 ºC.

- **Saliva (tube 5)**

1. Centrifuge at 1000 g for 5 minutes to separate the mucus.
2. Store the maximum volume of sample possible in a 2 ml cryotube.
3. Note the number of resulting aliquots, as well as their volume in the dataset, and store the aliquots at -80 ºC.

- **Urine (tube 6)**

1. Prepare three aliquots in 2 ml cryotubes.
2. Note the volume of each aliquot in the dataset and store at -80º C.

**Summary of samples collected:**

In total, 16 aliquots per patient should be obtained:

- 2 tubes with approximately 5 ml of whole blood + guanidine buffer. Store at 4ºC.
- 2 tubes with approximately 2 ml of plasma/glycerol and 1 tube with approximately 1 ml of plasma without glycerol. Store at -80ºC.
- 2 tubes with approximately 2 ml of serum/glycerol and 1 tube with approximately 1 ml of serum without glycerol. Store at -80ºC.
- 2 tubes with approximately 250 µl of heparinized blood. Store at -80ºC.
- 3 tubes with approximately 2 ml of saliva. Store at -80ºC.
- 3 tubes with approximately 2 ml of urine. Store at -80ºC.

**Proposed format for the sample storage dataset** (xls)

| **LABEL CODE** | **TYPE OF SAMPLE** | **DATE OF PROCESSING** | **VOLUME** | **LOCATION** | **RESPONSIBLE OF PROCESSING** | **OBSERVATIONS** |
| --- | --- | --- | --- | --- | --- | --- |
|  |  |  |  |  |  |  |
|  |  |  |  |  |  |  |
|  |  |  |  |  |  |  |
|  |  |  |  |  |  |  |
|  |  |  |  |  |  |  |

**Suggested label model**

| CHA 1-1111  __01/01/2022__  SUG |
| --- |
