# Supplementary material for: A guide for the generation of repositories of clinical samples for research on Chagas disease
Source: PLoS Negl Trop Dis. 2024 Aug 15;18(8):e0012166. doi: 10.1371/journal.pntd.0012166 (PMC11326570; doi:10.1371/journal.pntd.0012166)
Supplement: S3 File — (DOCX) [file pntd.0012166.s003.docx]

**Título: Guía para la generación de repositorios de muestras clínicas para la investigación de la enfermedad de Chagas.**

**Título corto: Guía para la generación de repositorios de muestras para la enfermedad de Chagas.**

**Autores:** Nieves Martínez-Peinado^1,2,*^, Juan Carlos Gabaldón-Figueira^1,*^, Roberto Rodrigues Ferreira^3,4^, María Carmen Thomas^5^, Manuel Carlos López^5^, Tania Cremonini Araújo-Jorge^3^, Belkisyolé Alarcón de Noya^6^, Soledad Berón^7^, Janine Ramsey^8^, Irene Losada Galván^1,9^, Alejandro G. Schijman^10^, Adriana González^11,12^, Andrés Mariano Ruiz^13,14^, Gimena Rojas^15^, Roberto Magalhães Saraiva^16^, Oscar Noya-Gonzalez^6,17,18^, Andrea Gómez^7^, Rosa A. Maldonado^19^, Jimmy Pinto^15^, Faustino Torrico^15^, Ivan Scandale^20^, Fernán Agüero^21,22^, María-Jesús Pinazo^20,23^, Joaquim Gascón^1,23^, Alejandro Marcel Hasslocher-Moreno^16^, Julio Alonso-Padilla^1,23,*^. En nombre de la Red NHEPACHA (Nuevas Herramientas para el Diagnóstico y Evaluación de la Enfermedad de Chagas)^**^

^*^Autor correspondiente

Correo electrónico: nieves.martinez@isglobal.org, juancarlos.gabaldon@isglobal.org julio.a.padilla@isglobal.org

^**^ Los miembros de la Red NHEPACHA se proporcionan en los agradecimientos

^1^Barcelona Institute for Global Health (ISGlobal), Hospital Clínic-University of Barcelona, Barcelona, Spain.

^2^Secció de Parasitologia, Departament de Biologia, Sanitat i Medi Ambient, Facultat de Farmàcia i Ciències de l'Alimentació, Universitat de Barcelona, 08007 Barcelona, Spain.

^3^Laboratory of Innovations in Therapies, Education and Bioproducts, Oswaldo Cruz Institute, Oswaldo Cruz Foundation (LITEB-IOC/Fiocruz), Brazil.

^4^Laboratory of Applied Genomics and Bioinnovations, Oswaldo Cruz Institute, Oswaldo Cruz Foundation, (LAGABI-IOC/Fiocruz) Rio de Janeiro, Brazil.

^5^ Instituto de Parasitología y Biomedicina López Neyra, Consejo Superior de Investigaciones Científicas (IPBLN-CSIC). PTS-Granada. Avda. del Conocimiento 17, 18016-Granada, Spain.

^6^Instituto de Medicina Tropical, Facultad de Medicina, Universidad Central de Venezuela, Caracas, Venezuela

^7^Fundación Mundo Sano

^8^Centro Regional de Investigación en Salud Pública (CRISP), Instituto Nacional de Salud Pública (INSP), Tapachula, Chiapas, México.

^9^Hospital Universitario 12 de Octubre, Madrid, Spain.

^10^Laboratorio de Biología Molecular de la Enfermedad de Chagas, Instituto de Investigaciones en Ingeniería Genética y Biología Molecular “Dr. Héctor N. Torres” - INGEBI-CONICET, Buenos Aires, Argentina.

^11^Departamento de Investigación, Salvando Latidos A.C., Guadalajara, Mexico

^12^Departamento de Investigación, Instituto Cardiovascular de Mínima Invasión (ICMI), Guadalajara, Mexico

^13^Instituto Nacional de Parasitología “Dr Mario Fatala Chaben” ANLIS MALBRÁN, Ministerio de Salud, Buenos Aires, Argentina.

^14^CONICET, Consejo Nacional de Investigaciones Científicas y Técnicas, Buenos Aires, Argentina.

^15^Universidad Mayor de San Simón and Fundación CEADES, Cochabamba, Bolivia.

^16^Evandro Chagas National Institute of Infectious Diseases, Oswaldo Cruz Foundation, Rio de Janeiro, Brasil.

^17^Cátedra de Parasitología, Escuela ¨Luís Razetti” Facultad de Medicina, Universidad Central de Venezuela, Caracas, Venezuela.

^18^Centro para Estudios Sobre Malaria, Instituto de Altos Estudios “Dr. Arnoldo Gabaldón”, Ministerio del Poder Popular para la Salud (MPPS), Caracas, Venezuela.

^19^Department of Biological Sciences, The University of Texas at El Paso, El Paso, Texas, USA.

^20^Drugs for Neglected Diseases Initiative (DND*i*), Geneva, Switzerland.

^21^Instituto de Investigaciones Biotecnológicas (IIB)–Consejo Nacional de Investigaciones Científicas y Técnicas (CONICET), San Martín, Buenos Aires, Argentina.

^22^Escuela de Bio y Nanotecnologías (EByN), Universidad de San Martín (UNSAM), San Martín, Buenos Aires, Argentina.

^23^CIBER de Enfermedades Infecciosas, Instituto de Salud Carlos III (CIBERINFEC, ISCIII), Madrid, Spain.

**Resumen**

La enfermedad de Chagas, causada por el parásito *Trypanosoma cruzi*, afecta a > 6 millones de personas en el mundo, principalmente en América Latina. Se distinguen dos fases clínicas principales en el curso de la misma: aguda y crónica. Actualmente, están disponibles dos fármacos anti-parasitarios para tratar la enfermedad (nifurtimox y benznidazol), pero los métodos diagnósticos requieren de equipamiento complejo y personal entrenado en su manejo, factores que dificultan su uso en amplias áreas de zonas endémicas, y el acceso al tratamiento de los pacientes.

Nuevas técnicas diagnósticas, como las pruebas rápidas (*RDTs*) para diagnosticar Chagas crónico o la amplificación isotérmica en bucle (*LAMP*), para detectar infecciones agudas, surgen como una alternativa valiosa, pero la diversidad genética del parásito podría dificultar su implementación. Además, determinar la eficacia del tratamiento para la enfermedad de Chagas es complicado, pues está determinada por la lenta reversión de la reactividad de los anticuerpos serológicos anti-*T. cruzi*, que incluso puede conllevar décadas en ocurrir. Por tanto, urge disponer de biomarcadores que permitan evaluar tempranamente la eficacia terapéutica, y de diagnósticos funcionales frente a la diversidad de genotipos circulantes.

Para llevar a cabo estudios que puedan responder a estas necesidades, colecciones multinacionales de muestras de individuos infectados por *T. cruzi*, de buena calidad, trazabilidad y con información clínico-epidemiológica asociada, son necesarias. Este trabajo es un manual para la elaboración de dichos repositorios siguiendo procesos estandarizados y uniformes, tomando en consideración los aspectos éticos, técnicos y logísticos del proceso. Puede ser adaptado según los recursos de cada laboratorio para la creación de colecciones de muestras clínicas obtenidas en condiciones homogéneas, favoreciendo el intercambio de información y material entre distintos grupos, así como la generalización de su evaluación y análisis. El objetivo es poder acelerar el desarrollo de nuevos métodos diagnósticos y la identificación de biomarcadores para la enfermedad de Chagas.

**Resumen del autor**

El diagnóstico de la enfermedad de Chagas requiere equipamiento costoso y personal capacitado, cuya falta dificulta el acceso al diagnóstico y tratamiento en vastas áreas de regiones endémicas. Además, la evaluación oportuna de la eficacia del tratamiento es complicada debido a lenta reversión de la serología anti-*T. cruzi*. Por tanto, existe una necesidad urgente de biomarcadores de eficacia terapéutica temprana y pronóstico de la enfermedad, así como herramientas diagnósticas más prácticas. Para realizar estudios que aborden estas necesidades, es fundamental contar con colecciones de muestras clínicas de buena calidad, trazabilidad e información clínico-epidemiológica asociada. En este trabajo, proporcionamos un protocolo estándar para recolectar, procesar, almacenar y transportar muestras clínicas de pacientes con la enfermedad de Chagas. El manual se elaboró tras alcanzar un consenso entre los expertos de la Red NHEPACHA, una coalición de investigadores clínicos y académicos de las Américas y España que busca la identificación y validación de nuevos biomarcadores y diagnósticos para la enfermedad de Chagas.

# **1. Introducción**

La enfermedad de Chagas, causada por el parásito protozoo *Trypanosoma cruzi* (*T. cruzi*), afecta a más de 6 millones de personas en todo el mundo y es la zoonosis parasitaria más importante de América Latina (1). Además, debido a importantes movimientos migratorios, en las últimas décadas se ha globalizado su impacto a nivel mundial, con la presencia de pacientes infectados en Europa, Norteamérica, Oceanía y Asia (2).

La enfermedad consta de una fase aguda y una fase crónica. La primera dura entre cuatro y ocho semanas, y es generalmente asintomática, aunque puede ser letal en un ~5% de los casos, particularmente en población pediátrica y personas inmunodeprimidas. La mayoría de los individuos infectados progresa hacia la fase crónica (3). Ésta puede prolongarse décadas a lo largo de las que el parásito persiste sin llegar a causar síntomas clínicos evidentes. No obstante, con el tiempo, aproximadamente un 30% de las personas infectadas crónicamente acaba desarrollando la sintomatología cardiaca y/o digestiva característica de la enfermedad (3).

Desde hace casi sesenta años existen dos fármacos antiparasitarios: benznidazol y nifurtimox. Ambos presentan buena eficacia al ser administrados en la fase aguda, pero ésta disminuye en la fase crónica, fase en la que se diagnostican la mayor parte de los casos. Además, ambos requieren de largos regímenes de administración que frecuentemente provocan efectos adversos, llegando a causar la interrupción del tratamiento. El diagnóstico precoz de la enfermedad es crucial para mejorar la eficacia del tratamiento, sin embargo, los síntomas en la fase aguda suelen ser inespecíficos, lo que dificulta su detección oportuna (1).

El diagnóstico de la fase crónica de la infección es indirecto y depende de la detección de inmunoglobulinas específicas anti-*T. cruzi*. Debido a la elevada diversidad antigénica del parásito, según recomendaciones de la Organización Mundial de la Salud (OMS) y la Organización Panamericana de la Salud (OPS), para obtener un diagnóstico confirmado de la infección es necesaria la concordancia entre dos técnicas serológicas basadas en distintos sets antigénicos (4). Sin embargo, las técnicas serológicas presentan ciertos inconvenientes como la posible reactividad cruzada con inmunoglobulinas contra otros parásitos estrechamente relacionados, como *Leishmania* spp., o la imposibilidad de calificar con ellas la eficacia del tratamiento pues la reversión a serología negativa ocurre en un periodo de tiempo tardío (4). Esto último es un inconveniente que dificulta, además, el desarrollo de ensayos clínicos para nuevas terapias contra la enfermedad. Además, realizar técnicas serológicas requiere de personal entrenado y equipamiento relativamente costoso, que hacen que no sean apropiadas en la mayoría de los centros de atención primaria de regiones endémicas.

En respuesta a esta problemática se desarrollaron los métodos inmunocromatográficos de diagnóstico rápido, conocidos como “*rapid diagnostic tests*” o RDTs (5,6). Son herramientas de diagnóstico fáciles de usar, no requieren de equipamiento eléctrico ni mantener cadena de frío en muestras o reactivos, y pueden proveer un resultado en menos de una hora. Aunque los RDTs se están utilizando para el tamizaje de la enfermedad en países como Bolivia, Colombia o Paraguay, las técnicas de serología convencional siguen siendo necesarias para confirmar el diagnóstico (6). Una alternativa que se ha propuesto el uso combinado de dos (o tres en caso de discordancia) RDTs basadas en sets antigénicos distintos (7−11). Si bien los RDTs han mostrado una alta sensibilidad y especificidad en algunas regiones con una alta prevalencia de infección (como ciertas áreas de Bolivia, el norte de Argentina y Colombia), su rendimiento es cuestionado en otras regiones geográficas, donde pueden predominar diferentes cepas de parásitos, así como en áreas de baja prevalencia de la infección (6, 12). Por tanto, el desarrollo de RDTs “universales” o adaptadas regionalmente con una amplia cobertura geográfica representa un objetivo muy atractivo**.**

Por otro lado, la falta de biomarcadores para la evaluación del pronóstico de la enfermedad y la valoración de la eficacia del tratamiento en la enfermedad de Chagas plantea un desafío significativo para los pacientes, los proveedores médicos y los investigadores clínicos. Dichos biomarcadores podrían identificar a aquellos con un mayor riesgo de daño orgánico y facilitar el desarrollo de nuevas pruebas para evaluar la eficacia terapéutica de los fármacos actualmente disponibles o los que están en ensayos clínicos (12−14).

Encontrar biomarcadores de respuesta al tratamiento y progresión de confianza supondría un hito en el campo. Diversas moléculas candidatas han sido evaluadas, pero la mayoría de estudios solo han incluido un limitado número de muestras clínicas de pacientes con periodos de seguimiento post-tratamiento limitados a apenas unos pocos años (12). Así como para las pruebas diagnósticas, se requieren cohortes más grandes, debidamente clasificadas y caracterizadas, de origen geográfico diverso, para abordad este problema. Estas cohortes deberían incluir controles no infectados e individuos con seroconversión después de períodos de seguimiento prolongados para identificar con confianza controles de cura positivos. Evaluar marcadores de respuesta terapéutica temprana requiere un seguimiento extendido de los participantes, idealmente durante cinco años o más después del tratamiento, para estudiar la progresión crónica de la enfermedad (13). Para que el análisis de las muestras siguiendo metodologías similares sea comparable en diferentes regiones/países, estas siempre deben obtenerse, procesarse y almacenarse siguiendo protocolos estandarizados y reproducibles, además de estar vinculadas a datos clínicos-epidemiológicos de calidad

Las muestras biológicas pueden ser almacenadas en biobancos y/o bio-repositorios. Si bien en la actualidad no existe un concepto universalmente aceptado de biobanco, se puede definir como un establecimiento público o privado, sin ánimo de lucro, que contiene muestras biológicas organizadas y con información clínica asociada, las cuales pueden ser utilizadas por terceros en proyectos de investigación biomédica o asistenciales. Los biobancos suelen constituir un espacio centralizado que recibe muestras de distintos bio-repositorios. A diferencia de los biobancos, los bio-repositorios son colecciones de muestras que pertenecen a un grupo de investigación particular para su uso en proyectos de investigación y que no pretenden ser cedidas a otros investigadores.

En este manual se describen los distintos aspectos a considerar en la creación y mantenimiento de una colección de muestras clínicas de la enfermedad de Chagas. La información detallada aquí está acorde con las guías BRISQ (“Bioespecimen Reporting for Improved Study Quality”) (15). La metodología descrita es fruto del consenso y la armonización de procedimientos alcanzados dentro de la Red NHEPACHA (“Nuevas Herramientas para el Diagnóstico y la Evaluación del Paciente con Enfermedad de Chagas”). Esta Red fue creada en 2011 con el objetivo de identificar y validar el uso de biomarcadores para la enfermedad de Chagas y consta de 18 grupos de investigación de 9 países. El manual que aquí se describe ha sido revisado y aprobado por el grupo de expertos de la Red. Su fin último es facilitar la generación de nuevas colecciones de muestras clínicas de la enfermedad de Chagas, así como mejorar las ya existentes para que puedan ser utilizadas en estudios multinacionales y multicéntricos tanto para la evaluación de nuevas técnicas diagnósticas como para la validación de biomarcadores tempranos de eficacia terapéutica y marcadores pronósticos.

# **2. Ética**

La extracción, procesamiento y utilización de las muestras se ha de realizar en total conformidad con la revisión actual de la Declaración de Helsinki (64ª Asamblea General, Fortaleza, Brasil, octubre 2013) y conforme a la correspondiente legislación respecto a la materia en la provincia y país en donde se colecten. Además, cumplir con la legislación europea, conocida por sus estrictas medidas de protección de datos, podría agilizar la transferencia de muestras para futuros proyectos de investigación.

Las muestras sólo deben ser obtenidas después de que el participante haya firmado un consentimiento informado (CI). El CI debe incluir el objetivo u objetivos de investigación y el uso que se dará a las mismas (actual y futuro, si es necesario). Tanto éste como el correspondiente protocolo de estudio deberán ser aprobados por un Comité de Ética de Investigación Clínica. Este comité velará que los proyectos se desarrollen siguiendo las normas éticas y legales vigentes, mientras que los investigadores son responsables de la trazabilidad de las muestras y la confidencialidad de cualquier dato derivado.

El material biológico y la información clínica asociada al mismo deben quedar integrados en la colección, y estar disponibles para aquellos proyectos de investigación que lo requieran previa aprobación por un comité científico. Las muestras biológicas se podrán conservar por el intervalo de tiempo marcado en el protocolo específico de estudio y CI, y asociados a las mismas deberán conservarse los datos clínicos de las personas donantes de las mismas. Tanto las muestras como sus datos deben ser sometidas a un proceso de anonimizado o codificación que será el utilizado por los investigadores para garantizar la protección de los mismos, y únicamente el personal autorizado podrá relacionar la identidad de los pacientes con dichos códigos. Idealmente, los datos clínicos y la información de las muestras biológicas deberán ser integrados en un fichero físico y electrónico del que será responsable cada centro de investigación. Dichos datos serán tratados y cedidos con la única y exclusiva finalidad de llevar a cabo la investigación biomédica tal y como se defina en los documentos del protocolo y CI del estudio que corresponda.

El uso retrospectivo de muestras con fines no revelados en el momento de la recogida de muestras debe estar de acuerdo con la información incluida en los formularios de consentimiento informado (IC). De lo contrario, se deberá obtener otro IC firmado por el participante. Por otro lado, solicitar muestras biológicas que ya han sido recolectadas, junto con su información clínico-epidemiológica asociada, plantea varios desafíos que deben ser considerados: procedimientos y pautas estrictas que abarcan permisos, documentación y cumplimiento de estándares éticos y regulatorios que pueden variar entre países.

# **3. Extracción de las muestras**

El tipo de muestra a colectar y cómo será su posterior procesado (Sección 4. Procesamiento de las muestras) estará definido por el tipo de ensayos que se vayan a realizar con las mismas. De preferencia, los participantes deben ser reclutados tan pronto como se confirme la infección por *T. cruzi*, tratando de garantizar su asistencia y continuidad en próximas visitas. En función del estudio, puede ser criterio de exclusión que el paciente haya recibido tratamiento, aunque no lo haya finalizado, como por ejemplo sería deseable en aquellos en los que se evalúe el rendimiento de nuevas herramientas de diagnóstico serológico (por ejemplo, RDTs). Es poco probable que se conozca el estado de infección de un individuo durante la primera visita. Incluso después de que se recolecten las primeras muestras, el diagnóstico puede tardar varias semanas, especialmente en áreas endémicas. También, la forma clínica del participante requerirá que se realicen exámenes (por ejemplo, electrocardiografía) y esa información deberá vincularse a la muestra recolectada.

La extracción de las muestras se debe realizar en un consultorio clínico o infraestructura médica que cuente la infraestructura apropiada. Los tiempos de recogida de muestras al seguimiento dependerán del resultado diagnóstico, determinado con la muestra de la primera visita. Normalmente, de acuerdo a la práctica clínica, en aquellas personas con la infección que cumplan con los requisitos médicos para recibir tratamiento anti-parasitario, se obtendrán muestras antes y después del tratamiento, seis meses tras el tratamiento y cada año tras la fecha de fin del mismo. En aquellas personas *T. cruzi* negativas, no infectadas, que no reciben tratamiento, se deberá tratar de conseguir muestras tras uno, cinco y diez años de la primera visita (de “reclutamiento”). En todos los casos, las muestras deben ser correctamente etiquetadas con la codificación correspondiente a cada paciente, la fecha de extracción y el tipo de muestra obtenida. La codificación de las muestras estará aparejada con la del laboratorio encargado del procesamiento. Debería permitirse cierta flexibilidad en el período de visitas (por ejemplo, 2-3 meses antes o después de la fecha programada) debido a las dificultades para mantener la agenda.

Una vez colectadas, las muestras idealmente transportadas a 4 ºC al laboratorio y almacenadas a esa temperatura hasta ser procesadas (Sección 4. Procesamiento de las muestras) en un tiempo máximo de 24 horas post-extracción. En la Figura 1, se muestra el circuito de recolección de muestras, su transporte, procesamiento y almacenamiento en el laboratorio.

**Figura 1. Circuito de recolecta de muestras en el consultorio clínico y posterior procesamiento en el laboratorio.**


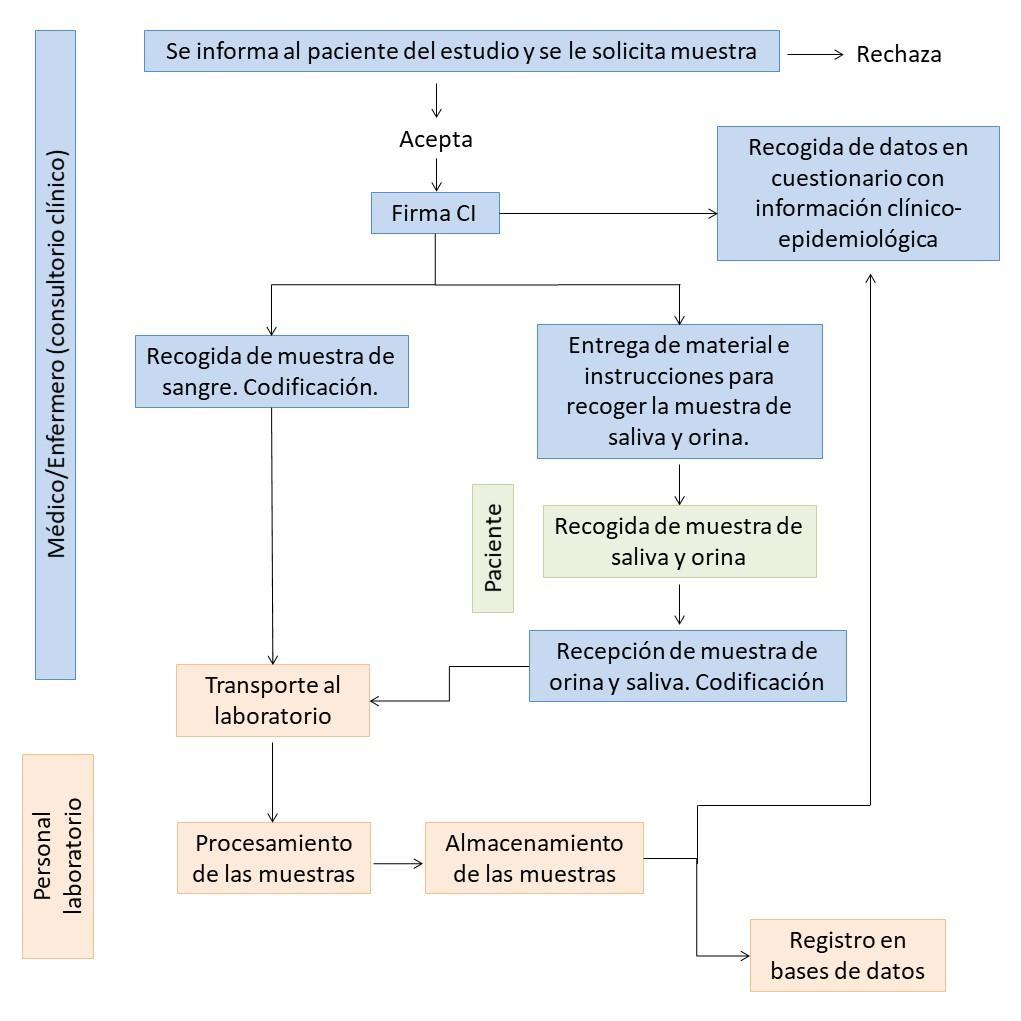


## **3.1. Extracción de sangre**

### **3.1.1 Procedimientos generales para la extracción de sangre**

La extracción de muestras de sangre debe ser realizada por personal entrenado para evitar la disconformidad del participante y no comprometer la calidad y/o cantidad de las muestras. Usualmente se realizan extracciones de sangre por punción venosa, utilizando ya sea aguja o jeringa o mediante un sistema de extracción al vació con tubos de plástico intercambiables (16). La extracción de sangre venosa puede realizarse según las técnicas habitualmente empleadas en cada institución. Sin embargo, es necesario seguir ciertas recomendaciones generales de flebotomía, puesto que una extracción incorrecta puede tener trascendencia sobre el posterior proceso analítico. Por ejemplo, hay que mantener al paciente sentado o tendido, desinfectar la zona de punción con alcohol isopropílico al 70%, utilizar un torniquete y mantener la presión en la herida por al menos dos minutos para facilitar la hemostasia (Archivo S4).

Para la construcción de una biblioteca de muestras clínicas, será necesario extraer al menos dos muestras: una en un tubo tratado con anticoagulante, y otra un en un tubo sin tratar (Figura 2). De cara a la homogeneización del proceso y en base a la disponibilidad de tubos con anticoagulante, el ácido etilendiaminotetraacético dipotásico (EDTA; generalmente EDTA-K2 por ser él más común) deben ser utilizados con el anticoagulante de preferencia si es posible.

### **3.1.2. Sangre total y fracciones de la sangre**

La sangre obtenida debe separarse, principalmente, en tres tipos de muestras: suero, sangre total, y plasma. Hay que destacar que es importante que los tubos de sangre sean recolectados en un orden específico para evitar la contaminación cruzada de aditivos, extrayendo en primer lugar el tubo sin conservantes ni anticoagulantes destinado a la obtención de suero y luego aquellos que llevan anticoagulantes.

1. **El suero** es la muestra más utilizada en la búsqueda de biomarcadores y la evaluación de técnicas diagnósticas. Se ha de recolectar en tubos sin conservantes ni anticoagulantes (tubo seco). Si es posible se recomienda utilizar tubos específicos para la segregación de suero que contienen un gel inerte que separa el suero de la sangre coagulada impidiendo su contaminación con componentes celulares.
2. La **sangre total**, es útil para la evaluación de RDTs o técnicas parasitológicas como el micrométodo (4). Es también el tipo de muestra más usado para la realización de diagnóstico molecular, ya sea por medio de la técnica de amplificación en cadena de la polimerasa (PCR; cualitativa o cuantitativa) o por amplificación isotérmica en bucle (LAMP) (17,18). Generalmente se colecta en tubos con anticoagulante (EDTA) que deberán ser conservados en congelación (idealmente a -80 ºC, alternativamente a -20 ºC). Si se quiere conservar las muestras de sangre total para uso de diagnóstico molecular a 4 ºC por tiempo prolongado, se les debe agregar hidrocloruro de guanidina 6M – EDTA 0.2 M (GE), pH 8.00 en una proporción volumen:volumen 1:1. Éste es en un muy buen preservante y asegura la viabilidad de las muestras de sangre total para métodos moleculares, incluso si se almacenan a temperatura ambiente, lo que representa una ventaja importante en zona endémica. Se pueden utilizar tubos con GE precargada para facilitar el manejo de muchas muestras, aunque de una manera u otra, el uso de guanidina tiene el inconveniente de que se trata de un reactivo costoso. En el Archivo S4 se detalla el protocolo de preparación de dicho reactivo.

Por otro lado, si las muestras se van a utilizar en la evaluación del test molecular LAMP basado en la reactividad del sustrato calceina (por ejemplo, el *T. cruzi*-LAMP desarrollado pro Eiken Chemical Co., Ltd, Tokio, Japón), resulta aconsejable no usar EDTA para anticoagular las muestras ya que puede interferir en la metodología de revelado de la reactividad (18). En este caso se pueden utilizar tubos con heparina, que es el anticoagulante de preferencia cuando se realiza micrométodo, un test parasitológico directo basado en microscopia.

Se ha demostrado que las técnicas LAMP y PCR tienen una sensibilidad y especificidad casi idéntica entre sí, y muy superior a la de aquel (19). Mientras que, en comparación con la PCR, el LAMP resulta mucho más sencillo de hacer y requiere mínimas infraestructuras de laboratorio, por lo que resultaría particularmente interesante para el diagnóstico de Chagas congénito.

1. El **plasma** se obtiene a partir de muestras recogidas en tubos con anticoagulante, preferiblemente EDTA, ya que este es el compuesto más comúnmente utilizado para la recolección de muestras de sangre total, lo que facilita los aspectos logísticos del proceso de recolección de muestras. Otros anticoagulantes con diferentes mecanismos de acción pueden comprometer la calidad de la muestra.

En caso de tener que priorizar entre obtener muestras de suero o plasma, se recomienda elegir el primero por su facilidad de obtención y mejor funcionamiento en diversos sistemas diagnósticos. Sin embargo, es importante considerar que el plasma, a diferencia del suero, contiene los factores de coagulación de la sangre, de interés en el estudio de ciertos biomarcadores relacionados con la activación de la cascada de la coagulación (20). De cualquier manera, se recomienda que ambos tipos de muestras (suero y/o plasma) se acompañen también de la correspondiente muestra de sangre total para poder realizar los estudios moleculares pertinentes.

### **3.1.3. Volumen de sangre extraído**

El volumen de sangre extraído debe ser acorde a la edad de los participantes. Idealmente a los participantes con edad ≥18 años, se les extraerán por punción venosa hasta un máximo de 10 mL de sangre en tubos con EDTA (5 mL en cada uno de los tubos) y 5 mL de sangre en tubos sin conservantes ni anticoagulantes 🡪15 mL de sangre total extraída. A los participantes con edad comprendida entre 5 y 18 años, se les extraerán 5 mL de sangre en tubos con EDTA (2,5 mL en cada tubo) y otros 5 mL de sangre en tubos sin conservantes ni anticoagulantes para el suero 🡪10 mL de sangre total extraída. A los niños con edades comprendidas entre los 2 y los 5 años se les extraerá la mínima cantidad de muestra posible, de acuerdo a las directrices pediátricas locales (21,22). Los recién nacidos y los niños menores de 2 años constituyen otro grupo de interés, principalmente para el estudio serológico dentro del algoritmo de diagnóstico de la enfermedad de Chagas congénita. La sangre de los recién nacidos se puede obtener mediante extracción venosa de la parte posterior de la mano al nacer o mediante la recogida de gotas de sangre del talón (alrededor de 500 µL). A partir de los nueve meses de edad, sería posible obtener alrededor de 1 mL de sangre mediante punción venosa en el brazo.

Además, se debe valorar colectar una fracción de la sangre extraída (250 a 500 µL) en tubos de heparina para poder realizar ensayos LAMP. Estos también podrían realizarse a partir de sangre seca colectada directamente sobre tarjetas de papel filtro como las de tipo FTA (por ejemplo QIAcard Flinders Technology Associates (FTA®)™ Classic cards (Qiagen, UK)) (23).

## **3.2. Extracción de orina y saliva**

El uso de técnicas serológicas para el diagnóstico de la infección por *T. cruzi* en muestras de orina (24) y saliva (25) representa un método de diagnóstico prometedor y poco invasivo. Por tanto, la inclusión de estas muestras en una colección clínica debería considerarse. Sin embargo, la utilidad de las muestras de orina y saliva es relativamente limitada en comparación con la sangre cuando es necesario priorizar, especialmente al considerar el espacio disponible y los costos asociados para mantener las muestras almacenadas. La recolección de orina es realizada por los propios participantes. Se deben suministrar instrucciones de forma verbal y escrita (Archivo S4). Aunque es posible que el participante recolecte la muestra de forma autónoma en su casa, es recomendable hacerlo en el centro de atención médica donde se están extrayendo las otras muestras, y durante la misma visita. Idealmente se han de recoger 5 - 10 mL de la primera orina de la mañana (26).

La colecta de saliva será realizada también en forma autónoma por los participantes (Archivo S4) preferiblemente en el mismo centro médico, igual que para el resto de muestras. Se requerirá que el paciente no beba, coma, fume, se lave los dientes o mastique chicle en la hora previa a su extracción, aunque sí se permite beber agua. Igualmente, se recogerán entre 5 y 10 mL de saliva.

# **4. Procesamiento de las muestras**

Todas las muestras humanas deben ser consideradas como potencialmente peligrosas por riesgo biológico, y por tanto, deberán ser procesadas en una cabina de bioseguridad de flujo laminar de tipo II (nivel de bioseguridad 2) para proteger al operador. Además, de esta forma se minimizan los riesgos de que las propias muestras puedan sufrir algún tipo de contaminación. Hay que resaltar que será clave que todos los tubos de muestras de laboratorio estén correctamente etiquetados antes de comenzar el procesamiento, con la codificación pertinente del paciente, el tipo de muestra y la fecha de procesamiento.

A pesar de requerir de mayor espacio de almacenamiento, resulta aconsejable hacer varias alícuotas de todas las muestras procesadas, por ejemplo tres, que idealmente se dispondrán en microtubos roscados con junta tórica para una mejor conservación a largo plazo. En caso de no ser posible se deberá tener al menos una doble alícuota. En laboratorios con problemas de almacenamiento cabe la posibilidad de realizar una alícuota única y sub-alicuotarla tras la primera descongelación, puesto que una única descongelación no afectará a su calidad. Posteriormente, se deberá registrar el número de veces que se ha descongelado la sub-alícuota de uso, puesto que este proceso de congelado y descongelado puede influir sobre la calidad de la muestra en función de la situación de almacenaje y lo que se pretenda medir en ella. Si la muestra contiene glicerol (previamente autoclavado y al menos 99% puro) en una proporción 1:1 este proceso puede realizarse hasta 10 veces sin que se vean afectados los anticuerpos presentes en la misma. Si la muestra no contiene glicerol, el proceso de descongelado-congelado no debe realizarse más de tres veces. En el Archivo S5 se muestra el procedimiento operativo estándar para el procesamiento de las siguientes muestras.

## **4.1. Suero**

Para la segregación del suero se debe centrifugar el tubo de sangre sin conservante a 1.600 g durante 10 min a temperatura ambiente. En ausencia de una centrífuga, es posible dejar coagular y precipitar la sangre por sí sola, pero es recomendable centrifugar para facilitar la separación siempre que sea posible. Como con las muestras de plasma (ver más abajo), se recomienda pipetear primero una cantidad de 2 a 3 mL, que al ser mezclada vol.:vol. 1:1 con glicerol resultaría en un volumen mayor que se debe distribuir en alícuotas de 1 a 2 mL en microtubos roscados. Este proceso es particularmente necesario en lugares donde el acceso a electricidad es irregular, puesto que esto puede comprometer la integridad de las muestras almacenadas. El uso de glicerol puede limitar aplicaciones posteriores de las muestras de suero, como por ejemplo para la evaluación de RDTs o el estudio de citoquinas, por lo que deberá tenerse en consideración procesar y preservar viales de suero con y sin glicerol.

## **4.2. Sangre total conservada en guanidina**

Para la obtención de sangre total, el volumen total (alrededor de 5 mL) de un tubo tratado con EDTA debe mezclarse con un volumen igual de GE (ref. G3272; Sigma-Aldrich). Tras mezclar bien por inversión, se divide el volumen resultante en dos tubos roscados con 5 mL cada uno. Conviene utilizar tubos con rosca externa, y con o-ring para evitar derrames durante el transporte. La guanidina es un agente caotrópico que lisa los eritrocitos y mantiene la integridad de los ácidos nucleicos.

## **4.3. Plasma**

Para la obtención de plasma se centrifuga el tubo de sangre con EDTA a 1.200 g durante 10 min a temperatura ambiente. Se recomienda obtener una cantidad de unos 2 ml tras centrifugar. Como para el suero, el volumen de plasma resultante puede mezclarse con una cantidad equivalente de glicerol. Como se discutió anteriormente, la producción de alícuotas sin glicerol puede ser necesaria dependiendo del objetivo con el que se colectaron las muestras. En ambos casos, se recomienda preparar alícuotas de 1 a 2 mL.

## **4.4. Saliva**

La muestra de saliva se debe centrifugar a 1.000 g durante 5 minutos a temperatura ambiente para eliminar la mucosidad, que queda en el pellet. Se tomará el sobrenadante, que deberá alicuotarse en microtubos roscados (de 1 a 2 mL por tubo).

## **4.5. Orina**

La orina no requiere ningún proceso de centrifugación. Sin embargo, es importante anotar las posibles alteraciones de la muestra (por ejemplo, un aumento de la turbidimetría por infección o hematuria) que podrían influir en el análisis posterior. Estas muestras también deben alicuotarse en microtubos roscados de 1 a 2 mL.

**Figura 2**. **Resumen de las diversas muestras clínicas generadas en un laboratorio de investigación clínica sobre enfermedad de Chagas.** Se muestra el número sugerido de alícuotas y volúmenes (Figura creada con Biorender; https://biorender.com/).


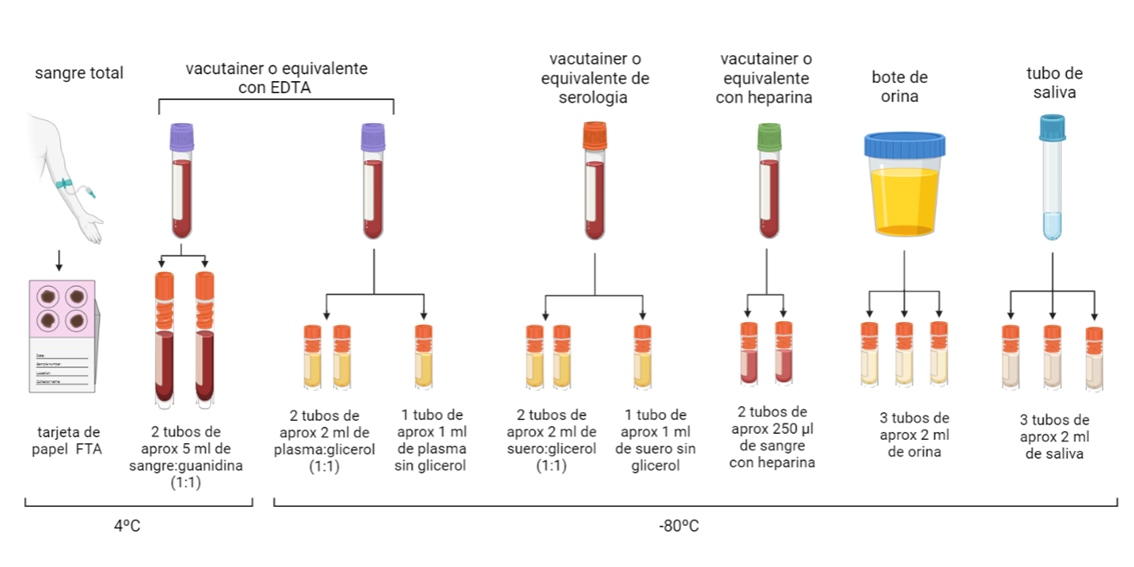


# **5. Almacenamiento**

Una vez procesadas y correctamente identificadas (se provee una hoja de etiquetado en el Archivo S5), es necesario registrar la localización y condiciones de almacenamiento de cada muestra obtenida. Esto incluye indicar cuántas veces se ha descongelado, en qué fecha y para qué estudio y/o análisis se ha utilizado. El número de descongelaciones ha de quedar registrado tanto en la alícuota como en la base de datos. En la Tabla 1 se detalla la temperatura ideal y aceptable a la que se deben conservar las muestras. En caso de no poder alcanzar una temperatura de -80 ºC, esta puede ser de hasta -70 ºC sin que afecte sobre la calidad de las muestras, ya que se sabe que es igual de eficaz y además contribuye al ahorro energético. Las muestras destinadas a ser utilizadas en estudios de producción de citoquinas siempre deben almacenarse a -70ºC o menos.

En zonas endémicas donde existan dificultades de suministro o equipamiento para alcanzar y mantener estas temperaturas, alternativamente se pueden conservar muestras de suero y plasma con azida sódica a 4 ºC durante largos periodos sin que se vea afectada su calidad. El azida sódica inhibe el crecimiento bacteriano y fúngico. Sin embargo, este procedimiento sólo es válido si las muestras serán usadas para análisis serológicos diagnósticos, y/o para conservar aquellas muestras (controles positivos) que se utilizan de rutina en estudios serológicos para evitar su descongelación-congelación. No es un método de conservación recomendado si las muestras son para el estudio de biomarcadores.

**Tabla 1. Temperaturas de almacenamiento a largo plazo de las muestras clínicas.**

| **Muestra** | **Temperatura ideal (ºC)** | **Temperatura aceptable (ºC)** |
| --- | --- | --- |
| **Sangre total – guanidina o sangre total en tarjetas FTA** | 4 | Temperatura ambiente |
| **Sangre total – EDTA o sangre total – heparina** | -80 | -20 |
| **Suero** | -80 | -20 |
| **Plasma** | -80 | -20 |
| **Saliva** | -80 | -20 |
| **Orina** | -80 | -20 |

# **6. Base de datos y aparejamiento de datos clínicos y de laboratorio**

Para poder ser utilizada, toda colección de muestras clínicas debe estar adecuadamente documentada en cuanto a la información clínico-epidemiológica que acompaña a cada muestra y contar con los consentimientos informados pertinentes. El CI utilizado para obtener las muestras también debe ser almacenado. Además, los cambios oportunos realizados en la misma (uso parcial de muestras, finalización de muestras,…) deben ser registrados en todo momento. En vista de estas entradas de información distintas pero relacionadas, es muy recomendable mantener todos los registros en un conjunto de datos centralizado cuyo nivel de sofisticación dependerá de los recursos del laboratorio y el volumen de muestras a manejar, yendo desde cuadernos de registro en papel a bases de datos informáticas o software multiusuario a tiempo real.

Estas bases de datos deben incluir la localización específica de las muestras y los datos clínicos y epidemiológicos. Para facilitar el proceso de recogida de datos es muy importante preparar cuestionarios para la captura, validación, almacenamiento y gestión de metadatos resultantes. Aunque estos cuestionarios pueden ser en papel, sería muy recomendable almacenar una copia digital de cada uno de ellos. En dichos cuestionarios (como el elaborado por González y colaboradores en paralelo a este documento) se registra la información clínica y epidemiológica, así como la información de laboratorio correspondiente a cada tipo de muestra, volumen y fecha de obtención y procesamiento, y cualquier posible incidencia relacionada con la misma.

# **7. Transporte de muestras**

Antes de transferir cualquier muestra de un centro a otro, se debe firmar un acuerdo entre ambas instituciones y cumplir con todos los requisitos exigidos por la ley. Entre esta documentación suele constar un acuerdo de transferencia de materiales y la documentación para la empresa de transporte entre la que se debe incluir una factura de aduanas, un listado de contenido del material y un permiso de importación en caso de ser necesario.

Una vez lista la documentación se puede proceder a preparar el paquete de envío. Para esto se debe asegurar que tanto el embalaje como el método de envío cumplan con las regulaciones aplicables. La institución que envía las muestras debe clasificarlas atendiendo a los criterios establecidos por las distintas agencias reguladoras de transporte.

Por otro lado, para preservar la calidad de las muestras se deberá registrar la temperatura y evitar sus fluctuaciones. Si se dispone de ello, se recomienda el uso de un data-logger para monitorizar la temperatura durante el transporte. Idealmente, las muestras de suero, plasma, orina y saliva se deben transportar congeladas con hielo seco asegurando una temperatura regular de unos -70 ºC. En tal caso, se ha de considerar que el hielo seco empleado en el envío está clasificado como material peligroso y se ha de etiquetar como tal (26). Si el transporte no puede realizarse a esta temperatura, puede hacerse a -20 ºC o refrigerado con packs de gel diseñados para ello o incluso a temperatura ambiente. En algunos casos, es aceptable realizar el transporte a temperatura ambiente, especialmente si las muestras fueron mezcladas con glicerol, aunque en tal caso se ha de considerar que las muestras ya se habrían descongelado una vez antes del envío.

En todos los casos, el paquete deberá ser lo suficientemente grande para contener las muestras y el método de refrigeración seleccionado. Habrá que considerar que siempre conviene colocar las muestras entre el refrigerante, en lugar de arriba o abajo del mismo para asegurar la estabilidad de la temperatura a la que están expuestas.

La institución que recibe las muestras es responsable de verificar el contenido de las mismas y las condiciones de llegada. Ante cualquier incidencia ha de comunicarlo a la institución que las envió.

# **8. Conclusión**

Esperamos que los procedimientos aquí descritos sirvan para guiar el establecimiento de nuevos y/o mejorados repositorios de muestras clínicas para el estudio de la enfermedad de Chagas; resultando clave la estandarización de un manejo de las mismas de calidad y trazabilidad, de forma que puedan alimentar la búsqueda de nuevos métodos diagnósticos y/o biomarcadores tanto de eficacia terapéutica como pronósticos. Se recomienda utilizar esta guía en combinación con la guía del cuestionario clínico (de González y colaboradores, enviado a revisión) para cada muestra biológica.

# **Agradecimientos**

Nos gustaría agradecer a DND*i* y Fundación Mundo Sano por su continuo apoyo a la Red NHEPACHA. MCT y MCL fueron apoyados por la Subvención PID2019-109090RB-100/AEI/10.13039/501100011033 del Programa Estatal I+D+I, Ministerio de Ciencia e Innovación de España (MICINN). Reconocemos el apoyo de la subvención CEX2018-000806-S financiada por MCIN/AEI/10.13039/501100011033. ILG, JCGF, NMP, JG y JAP reconocen el apoyo de la Generalitat de Catalunya a través del Programa CERCA. JCGF recibió apoyo a través de una beca de la Fundación "la Caixa" (ID 100010434, código de beca: LCF/BQ/DI21/11860037)

Grupo de estudio de la Red NHEPACHA:

Janine Ramsey , Angelica Pech May, Alba Valdez Tah, Gilberto Sanchez Gonzalez, Adriana Gonzalez Martinez, Eduardo Ortiz Panozo, Mario J. Grijalva, Jaime A. Costales, Cesar A. Yumiseva, Carolina Herrera, Eileen Velez, Maria de Lourdes Torres, Maria-Jesus Pinazo, Sergio Sosa Estani, Colin Forsyth, Eric Chatelain, Ivan Scandale, Fabiana Barreira, Tayná Marques, Marina Certo, Alejandro Hasslocher, Roberto Saraiva, Mauro Mediano, Andrea Silvestre, Sergio Xavier, Luiz Sangenis, Fernanda Mendes, Gilberto Sperandio da Silva, Andrea Costa, Henrique Veloso, Marcelo Holanda, Flavia Mazzoli, Paula Simplício da Silva, Tania Araujo, Mariana Wagabi, Luciana Garzoni, Constança Brito, Roberto Ferreira, Rita Machado, Raquel Aguiar, Marcelo Abril, Soledad Beron, Alejandro Schijman, Silvia Longhi, Arturo Muñoz-Calderón, Belkisyole Alarcon de Noya, Oscar Noya Gonzalez, Arturo Muñoz, Cecilia Colmenares, Ivan Mendoza, Zoraida Diaz, Raiza Ruiz, Ana Andreina Alviares, María Carmen Thomas, Manuel Carlos Lopez, Adriana Egui, Celia Benitez, Inmaculada Gómez, Francisco Macias Huete, Andres Mariano Ruiz, Rocio Rivero, Mónica Esteva, Margarita Bisio, Marisa Fernandez, Yolanda Hernandez, Julio Alonso Padilla, Joaquim Gascon, Irene Losada Galván, Nieves Martinez Peinado, Juan Carlos Gabaldon, María Gabriela Alvarez, Lococo Bruno, Laucella Susana, Flavio Andrés Tóman Conte, Dr. Enrique Morral, Maria Cecilia Albareda, Fernán Agüero, Emir Salas Sarduy, Alejandro Ricci, Leonel Bracco, Mercedes Didier Garnham, Alejandro Luquetti, Igor Almeida, Ester Sabino, Felipe Guhl, Faustino Torrico

# **Referencias**

1. WHO. Chagas disease (American trypanosomiasis). [Cited 31 Jul 2023]. Available from: https://www.who.int/en/news-room/fact-sheets/detail/chagas-disease-(american-trypanosomiasis).

2. Alonso-Padilla J, Cortés-Serra N, Pinazo MJ, Elena M, Abril M, Barreira F, et al. Strategies to enhance access to diagnosis and treatment for Chagas disease patients in Latin America. Expert Rev Anti Infect Ther. 2019;17:145–57. doi: 10.1080/14787210.2019.1577731

3. Pinazo MJ, Gascon J. Chagas disease: from Latin America to the world. Reports Parasitol. 2015;4:7–14.

4. Gállego M, Schijman AG, Alonso-Padilla J. Diagnosis of Trypanosoma cruzi infection: challenges on laboratory tests development and applications. In: Pinazo MJ, Gascon J., editors. Chagas disease. A neglected tropical disease. Springer Nature; 2020, pp. 75–94.

5. Pinazo MJ, Gascon J, Alonso-Padilla J. How effective are rapid diagnostic tests for Chagas disease? Expert Rev Anti Infect Ther. 2021;19:1489-1494. doi: 10.1080/14787210.2021.1873130

6. Lozano D, Rojas L, Méndez S, Casellas A, Sanz S, Ortiz L, et al. Use of rapid diagnostic tests (RDTs) for conclusive diagnosis of chronic Chagas disease - field implementation in the Bolivian Chaco region. PLoS Negl Trop Dis. 2019;13:e0007877. doi: 10.1371/journal.pntd.0007877

7. Egüez KE, Alonso-Padilla J, Terán C, Chipana Z, García W, Torrico F, et al. Rapid diagnostic tests duo as alternative to conventional serological assays for conclusive Chagas disease diagnosis. PLoS Negl Trop Dis. 2017;11:e0005501. doi: 10.1371/journal.pntd.0005501

8. Mendicino D, Colussi C, Moretti E. Simultaneous use of two rapid diagnostic tests for the diagnosis of Chagas disease. Trop Doct. 2019;49:23-26. doi: 10.1177/0049475518813792

9. Lopez-Albizu C, Danesi E, Piorno P, Fernandez M, García Campos F, Scollo K, Crudo F. Rapid diagnostic tests for Trypanosoma cruzi infection: field evaluation of two registered kits in a region of endemicity and a region of nonendemicity in Argentina. J Clin Microbiol. 2020;58:e01140-20. doi: 10.1128/JCM.01140-20

10. Suescún-Carrero SH, Tadger P, Sandoval Cuellar C, Armadans-Gil L, Ramírez López LX. Rapid diagnostic tests and ELISA for diagnosing chronic Chagas disease: systematic revision and meta-analysis. PLoS Negl Trop Dis. 2022;16:e0010860. doi: 10.1371/journal.pntd.0010860

11. Angheben A, Buonfrate D, Cruciani M, Jackson Y, Alonso-Padilla J, Gascon J, et al. Rapid immunochromatographic tests for the diagnosis of chronic Chagas disease in at-risk populations: a systematic review and meta-analysis. PLoS Negl Trop Dis. 2019;13:e0007271. doi: 10.1371/journal.pntd.0007271

12. Cortes-Serra N, Losada-Galvan I, Pinazo MJ, Fernandez-Becerra C, Gascon J, Alonso-Padilla J. State-of-the-art in host-derived biomarkers of Chagas disease prognosis and early evaluation of anti-Trypanosoma cruzi treatment response. Biochim Biophys Acta Mol Basis Dis. 2020;1866:165758. doi: 10.1016/j.bbadis.2020.165758.

13. Pinazo MJ, Thomas MC, Bua J, Perrone A, Schijman AG, Viotti RJ, et al. Biological markers for evaluating therapeutic efficacy in Chagas disease, a systematic review. Expert Rev Anti Infect Ther. 2014;12:479-96. doi: 10.1586/14787210.2014.899150

14. Mendes VG, Rimolo L, de Lima ACB, Ferreira RR, Oliveira LS, Nisimura LM, et al. Biomarkers and echocardiographic predictors of cardiovascular outcome in patients with chronic Chagas disease. J Am Heart Assoc. 2023;12(12):e028810. doi: 10.1161/JAHA.122.028810.

15. Moore HM, Kelly AB, Jewell SD, McShane LM, Clark DP, Greenspan R, et al. Biospecimen reporting for improved study quality (BRISQ). Cancer Cytopathol. 2011;119:92-101. doi: 10.1002/cncy.20147

16. Vaught JB, Henderson MK. Biological sample collection, processing, storage and information management. IARC Sci Publ. 2011;23–42.

17. Muñoz-Calderón AA, Besuschio SA, Wong S, Fernández M, García Cáceres LJ, Giorgio P, et al. Loop-mediated isothermal amplification of Trypanosoma cruzi DNA for point-of-care follow-up of anti-parasitic treatment of Chagas disease. Microorganisms. 2022;10:909. doi: 10.3390/microorganisms10050909

18. Polley SD, González IJ, Mohamed D, Daly R, Bowers K, Watson J, et al. Clinical evaluation of a loop-mediated amplification kit for diagnosis of imported malaria. J Infect Dis. 2013;208:637-44. doi: 10.1093/infdis/jit183

19. Besuschio SA, Llano Murcia M, Benatar AF, Monnerat S, Cruz I, Picado A, et al. Analytical sensitivity and specificity of a loop-mediated isothermal amplification (LAMP) kit prototype for detection of Trypanosoma cruzi DNA in human blood samples. PLoS Negl Trop Dis. 2017;11:e0005779. doi: 10.1371/journal.pntd.0005779

20. Pinazo MJ, Posada Ede J, Izquierdo L, Tassies D, Marques AF, de Lazzari E, et al. Altered hypercoagulability factors in patients with chronic Chagas disease: potential biomarkers of therapeutic response. PLoS Negl Trop Dis. 2016;10:e0004269. doi: 10.1371/journal.pntd.0004269

21. Martorell LLCM. Hemocultivos en el Instituto de Hematología e Inmunología: optimizando la toma de muestra. Rev Cuba Hematol Inmunol y Hemoter. 2019;37:1–16.

22. Calvo Cillán A. Utilidad de la extracción de un volumen adecuado de sangre para aumentar la rentabilidad de los hemocultivos en pediatría. M. Sc. Thesis. Universidad Internacional de Andalucía. 2018. Available from: https://dspace.unia.es/bitstream/handle/10334/3889/0857_Calvo.pdf?sequence=1&isAllowed=y

23. Longhi SA, García Casares LJ, Muñoz-Calderón AA, Alonso-Padilla J, Schijman AG. Combination of ultra-rapid DNA purification (PURE) and loop-mediated isothermal amplification (LAMP) for rapid detection of Trypanosoma cruzi DNA in dried blood spots. PLoS Negl Trop Dis. 2023;17(4):e0011290. doi:10.1371/journal.pntd.0011290

24. Castro-Sesquen YE, Gilman RH, Galdos-Cardenas G, Ferrufino L, Sánchez G, Valencia Ayala E, et al. Use of a novel Chagas urine nanoparticle test (chunap) for diagnosis of congenital Chagas disease. PLoS Negl Trop Dis. 2014;8:e3211. doi: 10.1371/journal.pntd.0003211

25. Cortes-Serra N, Pinazo MJ, de la Torre L, Galizzi M, Gascon J, Bustamante JM. Diagnosis of Trypanosoma cruzi infection status using saliva of infected subjects. Am J Trop Med Hyg. 2018;98:464-467. doi: 10.4269/ajtmh.17-0141.

26. Campbell LD, Astrin JJ, DeSouza Y, Giri J, Patel AA, Rawley-Payne M, Rush A, Sieffert N. Best practices: recommendations for repositories. 4st ed. Vancouver: ISBER International Society for Biological and Environmental Repositories; 2018.
