# Supplementary material for: A guide for the generation of repositories of clinical samples for research on Chagas disease
Source: PLoS Negl Trop Dis. 2024 Aug 15;18(8):e0012166. doi: 10.1371/journal.pntd.0012166 (PMC11326570; doi:10.1371/journal.pntd.0012166)
Supplement: S4 File — (DOCX) [file pntd.0012166.s004.docx]

**S4. Procedimiento Operativo Estándar de Extracción de muestras clínicas para la generación de repositorios de la enfermedad de Chagas**

**1. Extracción de sangre**

**Material necesario:**

- Bandeja.
- Algodón.
- Alcohol.
- Smarch o goma para torniquete.
- Guantes de látex.
- Soporte y agujas para tubos con vacío (tipo vacutainer).
- Gradilla para tubos.
- 1 tubo de toma de muestra sin anticoagulante.
- 2 tubos con EDTA-K2.
- Esparadrapo (adhesivo) hipoalérgico o tirita.
- Marcador indeleble.
- Recipientes para desechar el material biológico. Etiquetas identificativas.

**Procedimiento de toma de muestras de sangre venosa:**

1. Verifique la identidad del paciente y la orden de análisis. Recuerde que en primer lugar se han de tomar los tubos sin anticoagulantes ni aditivos para evitar la contaminación cruzada.
2. Explicar el procedimiento al paciente.
3. Prepare el material de venopunción: goma para el torniquete, algodón, alcohol, esparadrapo, soporte y aguja tipo vacutainer y guantes para la toma de muestra.
4. Prepare e identifique con el código del paciente y la fecha los tubos que serán necesarios de acuerdo a los análisis requeridos. Utilice etiquetas para identificar los tubos.
5. Prepare la aguja vacutainer en su soporte y coloque la goma del torniquete con suficiente presión pero sin excederse, ya que si está muy apretado, produce hemólisis, colapso venoso, dolor y la muestra puede resultar inapropiada.
6. Palpe la vena del área antecubital que le parezca más apropiada, desinfecte con alcohol, deje secar el alcohol y pinche con el bisel de la aguja hacia arriba y formando un ángulo de aproximadamente 15^o^.
7. En caso de colectar muestra sobre tarjetas de papel FTA, deje caer unas gotas directamente sobre el papel. Llene los círculos del papel FTA transfiriendo una gota de sangre por cada círculo. Los círculos de sangre deben ser simétricos en ambos lados y deben llenar correctamente el área marcada.
8. Introduzca el papel FTA en una bolsa metálica con sílica gel. Conserve a 4 ºC hasta su transporte al laboratorio.
9. Presione el tubo de vacío sobre el extremo de la aguja que se encuentra dentro del dispositivo y deje que se llene. Una vez lleno, saque el tubo del dispositivo e introduzca el siguiente. Recuerde que en primer lugar se ha de extraer el tubo sin conservantes ni anticoagulantes para evitar la posible contaminación cruzada de aditivos.
10. Retire el último tubo del soporte de la aguja, retire el torniquete, retire la aguja y presione firmemente con un algodón el lugar de punción.
11. Deseche la aguja en el recipiente de material cortopunzante.
12. Los tubos con anticoagulante deben llenarse hasta que se acabe el vacío para mantener una proporción adecuada entre la muestra y el anticoagulante. Deben mezclarse inmediatamente por inversión suavemente entre 5 y 10 veces para que no se coagule la sangre.
13. Verifique que ha tomado todas las muestras requeridas en la orden de análisis; si todo está completo, el paciente puede retirarse.
14. Mantenga las muestras refrigeradas a 4 ºC hasta su transporte al laboratorio. El procesamiento debe realizarse dentro de las 24 horas post-extracción.

**2. Extracción de saliva**

**Material necesario:**

- Tubo de recolección de saliva.

**Procedimiento de toma de muestras de saliva:**

Se le han de suministrar las siguientes instrucciones al paciente de forma oral y escrita.

1. No comer, masticar chicle, beber o fumar durante una hora previa a la toma de la muestra. Si se permite beber agua. Para evitar que la muestra se contamine evite el uso de pintalabios, cremas, o inhaladores que contengan esteroides antes de la recogida de la muestra. No haga ninguna actividad que pueda provocar sangrado de las encías, como el cepillado de los dientes; ni utilice estimulantes salivares como ácido ascórbico, ni elixires bucales.
2. Frote sus mejillas y paladar con la lengua para la estimulación de la secreción de saliva.
3. Escupa la saliva en el tubo hasta que el líquido (sin incluir las burbujas) alcance la línea de 5-10 mL en el tubo de recolección. El exceso de burbujas se puede eliminar golpeando suavemente el tubo contra una superficie.
4. Cierre el tubo de recolección de saliva.

**3. Extracción de orina**

**Material necesario:**

- Tubo de recolección de orina.

**Procedimiento de toma de muestras de orina:**

Se le han de suministrar las siguientes instrucciones al paciente de forma oral y escrita.

El paciente deberá:

1. Lavarse las manos.
2. Separar bien los labios mayores con la mano o retirar el prepucio y comenzar a orinar, desechando el primer chorro de orina.
3. A continuación, orine en el frasco estéril evitando que el chorro de orina toque la piel de las manos, o genitales.
4. Cierre el tubo evitando tocar el interior del mismo.
