# Supplementary material for: A guide for the generation of repositories of clinical samples for research on Chagas disease
Source: PLoS Negl Trop Dis. 2024 Aug 15;18(8):e0012166. doi: 10.1371/journal.pntd.0012166 (PMC11326570; doi:10.1371/journal.pntd.0012166)
Supplement: S5 File — (DOCX) [file pntd.0012166.s005.docx]

**S5. Procedimiento Operativo Estándar de procesamiento de muestras clínicas para la generación de repositorios de la enfermedad de Chagas**

**Resumen de la toma de muestras:**

|  | **Sangre total (aprox. 15 mL)** |
| --- | --- |
| **Condiciones** | - 10 mL colectados en 2 tubos EDTA (tapa lila). - 5 mL colectados en tubo “seco” sin conservante, con gel pro-coagulante (tapa roja). - 500 µL colectados en tubo con heparina (tapa verde). |
| **Conservación hasta recogida por personal laboratorio** | Refrigerador  4 ºC. |
| **Transporte** | Definir quién recogerá y transportará las muestras y en qué intervalo de tiempo (máx. 24h post-extracción). |

**Notas generales:**

- El procesamiento de todas las muestras se realizará en cabina de bioseguridad de nivel 2 (NSB2).
- Junto con las muestras biológicas se adjuntan todas las etiquetas para los tubos de muestras de laboratorio. Una vez etiquetados los tubos, cubrir las etiquetas con cinta adhesiva transparente para evitar su degradación.
- Al final del procesamiento, completar la base de datos de la colección.

**Reactivos:**

- Guanidina (6 M)- EDTA (0.2 M) pH 8.00: Guanidine hydrochloride, for molecular biology, ≥99%. MW: 95.53 (ref. G3272; Sigma). Para preparar Guanidine hydrochloride 6 M mezclar 100 gr guanidine hydrochloride + 174,5 mL H_2_O (ref. W4502; Sigma) y filtrar con filtro de 0.22 µm.
- Glicerol (pureza del 99%) (ref. G5516; Sigma): autoclavar.
- H_2_O_MQ_: autoclavar.

**Materiales:**

- Pipetas serológicas 5 mL.
- Tubos Falcon 15 mL.
- Microtubo roscado 2 mL.
- Criotubo roscado 5 mL.
- Tubo 4 mL EDTA k2 (tapa lila).
- Tubo 10 mL con gel actividad coagulante (tapa roja).

**Protocolo:**

1. Etiquetar todos los tubos de muestra que se van a generar en el laboratorio y cubrirlos con cinta adhesiva transparente.

Nota 1: realizar el etiquetado antes de comenzar el procesamiento puede evitar confusiones cuando se procesen varias muestras a la vez.

Nota 2: los volúmenes de sangre que se describen en el protocolo corresponden a muestras de pacientes ≥ 18 años. En caso de que la muestra sea de un paciente ≤ 18 años el volumen de muestra recibido será inferior, pero se ha de seguir el mismo procedimiento.

- **Sangre total (10 mL) colectada en 2 vacutainer o equivalente con EDTA (tapa lila):** a partir de esta muestra biológica se guardan dos tipos de muestras en laboratorio:
- **Sangre total + guanidina (1):**

1. Extraer el volumen de sangre total con EDTA de uno de los vacutainer en un tubo Falcon de 15 mL.
2. Añadir el mismo volumen de hidrocloruro de guanidina 6 (M) – EDTA (0.2 M), pH 8.00, de forma que quede en una proporción 1:1.
3. Mezclar por inversión y dividir el volumen en 2 tubos roscados de 5 mL.
4. Anotar el volumen en la base de datos. Conservación a 4 ºC.

- **Plasma (2):**

1. Centrifugar el vacutainer restante de sangre total con EDTA a 1.200 g durante 10 minutos a temperatura ambiente.
2. Extraer el máximo volumen de plasma en un tubo Falcon de 15 mL.
3. Extraer 1/3 del volumen obtenido y alicuotarlo en un microtubo roscado de 2 mL.
4. Añadir glicerol estéril a los 2/3 de plasma restantes en el Falcon de 15 mL, en una proporción 1:1.
5. Mezclar pipeteando arriba y abajo hasta tener una mezcla homogénea.
6. Dividir el volumen resultante en 2 alícuotas de idéntico volumen en microtubos roscados de 2 mL.
7. Anotar el volumen de cada una de las alícuotas en la base de datos. Guardar a -80 ºC.

- **Sangre total en vacutainer de serología (tapa roja) o equivalente, para la obtención de suero (3):**

1. Centrifugar el vacutainer a 1.600 g durante 10 minutos a temperatura ambiente.

Nota: por las propiedades del tubo, puede dejarse coagular y precipitar por sí solo, pero es mejor centrifugar.

1. Extraer el máximo volumen de suero en un tubo Falcon de 15 mL.
2. Extraer 1/3 del volumen obtenido y alicuotarlo en un microtubo roscado de 2 mL.
3. Añadir glicerol estéril a los 2/3 de plasma restantes en el Falcon de 15 mL, en una proporción 1:1.
4. Mezclar pipeteando arriba y abajo hasta tener una mezcla homogénea.
5. Dividir el volumen resultante en 2 alícuotas de idéntico volumen en microtubos roscados de 2 mL.
6. Anotar el volumen de cada una de las alícuotas en la base de datos. Guardar a -80 ºC.

- **Sangre total en vacutainer o equivalente con heparina, para la realización de ensayos LAMP (4):**

1. Hacer 2 alícuotas de 250 µL.
2. Anotar el volumen de cada una en la base de datos. Guardar a -80 ºC.

- **Saliva (5):**

1. Centrifugar a 1.000 g durante 5 minutos, para eliminar la mucosidad.
2. Guardar el máximo de muestra posible teniendo en cuenta el volumen máximo del criotubo (2 mL).
3. Anotar el volumen de cada una en la base de datos. Guardar a -80 ºC.

- **Orina (6):**

1. Hacer 3 alícuotas de 2 mL.
2. Anotar el volumen de cada una en la base de datos. Guardar a -80 ºC.

**Resumen de muestras generadas** (16 tubos finales (alícuotas totales) por paciente):

- 2 tubos de aprox 5 mL sangre total + buffer guanidina. Conservar a 4 ºC.
- 2 tubos de aprox. 2 mL con plasma en glicerol y 1 tubo de aprox 1 mL de plasma sin glicerol. Conservar a -80 ºC.
- 2 tubos de aprox. 2 mL de suero en glicerol y 1 tubo de aprox 1 mL de suero sin glicerol. Conservar a -80 ºC.
- 2 tubos de aprox. 250 µL de sangre-heparina. Conservar a -80 ºC.
- 3 tubos de aprox. 2 mL de saliva. Conservar a -80 ºC.
- 3 tubos de aprox. 2 mL de orina. Conservar a -80 ºC.

**Propuesta de base de datos para muestras de laboratorio (tipo xls):**

| **CÓDIGO ETIQUETA** | **TIPO DE MUESTRA** | **FECHA DE PROCESADO** | **VOLUMEN** | **LOCALIZACIÓN** | **PROCESADO POR:** | **OBSERVACIONES** |
| --- | --- | --- | --- | --- | --- | --- |
|  |  |  |  |  |  |  |
|  |  |  |  |  |  |  |
|  |  |  |  |  |  |  |
|  |  |  |  |  |  |  |
|  |  |  |  |  |  |  |

**Sugerencia modelo de etiquetas:**

| CHA 1-1111  __01/01/2022__  SUG |
| --- |
