# Supplementary material for: A guide for the generation of repositories of clinical samples for research on Chagas disease
Source: PLoS Negl Trop Dis. 2024 Aug 15;18(8):e0012166. doi: 10.1371/journal.pntd.0012166 (PMC11326570; doi:10.1371/journal.pntd.0012166)
Supplement: S6 File — (DOCX) [file pntd.0012166.s006.docx]

**Título: Um guia para geração de repositórios de amostras clínicas para pesquisa em doença de Chagas**

**Título curto: Guia para a geração de repositórios de amostras para a doença de Chagas.**

**Autores:** Nieves Martínez-Peinado^1,2,^*, Juan Carlos Gabaldón-Figueira^1,^*, Roberto Rodrigues Ferreira^3,4^, María Carmen Thomas^5^, Manuel Carlos López^5^, Tania Cremonini Araújo-Jorge^3^, Belkisyolé Alarcón de Noya^6^, Soledad Berón^7^, Janine Ramsey^8^, Irene Losada Galván^1,9^, Alejandro G. Schijman^10^, Adriana González^11,12^, Andrés Mariano Ruiz^13,14^, Gimena Rojas^15^, Roberto Magalhães Saraiva^16^, Oscar Noya-Gonzalez^6,17,18^, Andrea Gómez^7^, Rosa A. Maldonado^19^, Jimmy Pinto^15^, Faustino Torrico^15^, Ivan Scandale^20^, Fernán Agüero^21,22^, María-Jesús Pinazo^20,23^, Joaquim Gascón^1,23^, Alejandro Marcel Hasslocher-Moreno^16^, Julio Alonso-Padilla^1,23,^*. Em nome da Rede NHEPACHA (Novas Ferramentas para o Diagnóstico e Avaliação da Doença de Chagas)^**^

^*^Autor correspondente

Email: nieves.martinez@isglobal.org, juancarlos.gabaldon@isglobal.org julio.a.padilla@isglobal.org

^**^ A filiação à Rede NHEPACHA é fornecida nos agradecimentos

^1^Barcelona Institute for Global Health (ISGlobal), Hospital Clínic-University of Barcelona, Barcelona, Spain.

^2^Secció de Parasitologia, Departament de Biologia, Sanitat i Medi Ambient, Facultat de Farmàcia i Ciències de l'Alimentació, Universitat de Barcelona, 08007 Barcelona, Spain.

^3^Laboratory of Innovations in Therapies, Education and Bioproducts, Oswaldo Cruz Institute, Oswaldo Cruz Foundation (LITEB-IOC/Fiocruz), Brazil.

^4^Laboratory of Applied Genomics and Bioinnovations, Oswaldo Cruz Institute, Oswaldo Cruz Foundation, (LAGABI-IOC/Fiocruz) Rio de Janeiro, Brazil.

^5^ Instituto de Parasitología y Biomedicina López Neyra, Consejo Superior de Investigaciones Científicas (IPBLN-CSIC). PTS-Granada. Avda. del Conocimiento 17, 18016-Granada, Spain.

^6^Instituto de Medicina Tropical, Facultad de Medicina, Universidad Central de Venezuela, Caracas, Venezuela

^7^Fundación Mundo Sano

^8^Centro Regional de Investigación en Salud Pública (CRISP), Instituto Nacional de Salud Pública (INSP), Tapachula, Chiapas, México.

^9^Hospital Universitario 12 de Octubre, Madrid, Spain.

^10^Laboratorio de Biología Molecular de la Enfermedad de Chagas, Instituto de Investigaciones en Ingeniería Genética y Biología Molecular “Dr. Héctor N. Torres” - INGEBI-CONICET, Buenos Aires, Argentina.

^11^Departamento de Investigación, Salvando Latidos A.C., Guadalajara, Mexico

^12^Departamento de Investigación, Instituto Cardiovascular de Mínima Invasión (ICMI), Guadalajara, Mexico

^13^Instituto Nacional de Parasitología “Dr Mario Fatala Chaben” ANLIS MALBRÁN, Ministerio de Salud, Buenos Aires, Argentina.

^14^CONICET, Consejo Nacional de Investigaciones Científicas y Técnicas, Buenos Aires, Argentina.

^15^Universidad Mayor de San Simón and Fundación CEADES, Cochabamba, Bolivia.

^16^Evandro Chagas National Institute of Infectious Diseases, Oswaldo Cruz Foundation, Rio de Janeiro, Brasil.

^17^Cátedra de Parasitología, Escuela ¨Luís Razetti” Facultad de Medicina, Universidad Central de Venezuela, Caracas, Venezuela.

^18^Centro para Estudios Sobre Malaria, Instituto de Altos Estudios “Dr. Arnoldo Gabaldón”, Ministerio del Poder Popular para la Salud (MPPS), Caracas, Venezuela.

^19^Department of Biological Sciences, The University of Texas at El Paso, El Paso, Texas, USA.

^20^Drugs for Neglected Diseases Initiative (DND*i*), Geneva, Switzerland.

^21^Instituto de Investigaciones Biotecnológicas (IIB)–Consejo Nacional de Investigaciones Científicas y Técnicas (CONICET), San Martín, Buenos Aires, Argentina.

^22^Escuela de Bio y Nanotecnologías (EByN), Universidad de San Martín (UNSAM), San Martín, Buenos Aires, Argentina.

^23^CIBER de Enfermedades Infecciosas, Instituto de Salud Carlos III (CIBERINFEC, ISCIII), Madrid, Spain.

**Resumo:**

A doença de Chagas, causada pelo parasita *Trypanosoma cruzi*, afeta mais de 6 milhões de pessoas e seu impacto maior é concentra na América Latina. Duas fases clínicas, aguda e crônica, são reconhecidas. Atualmente, existem dois medicamentos anti-parasitários disponíveis para tratar a doença (nifurtimox e benznidazol), mas os métodos diagnósticos exigem uma infraestrutura relativamente complexa e equipe treinada, limitando seu uso generalizado em áreas endêmicas e o acesso dos pacientes ao tratamento.

Novos métodos de diagnósticos, como testes de diagnósticos rápidos (RDTs) para diagnosticar a doença de Chagas crônica, ou amplificação isotérmica mediada por loop (LAMP), para detecção de infecções agudas, representam alternativas valiosas, mas a notável diversidade genética do parasita pode dificultar sua implementação. Além disso, determinar a eficácia do tratamento da doença de Chagas, dada o lento desaparecimento da reatividade dos anticorpos anti-*T. cruzi* no soro, que pode levar até décadas para ocorrer. Novos biomarcadores para avaliar a eficácia terapêutica precoce, bem como testes diagnósticos capazes de detectar a grande variedade de genótipos circulantes são, portanto, urgentemente necessários.

Para realizar estudos que atendam a essas necessidades, amostras de alta qualidade e rastreáveis de indivíduos infectados por *T. cruzi* com diferentes origens geográficas, registradas com dados clínicos e epidemiológicos relevantes, são necessários. Este trabalho descreve uma estrutura para a criação de tais repositórios, seguindo protocolos padronizados e uniformes, e considerando os aspectos éticos, técnicos e logísticos do processo. O manual pode ser adaptado de acordo com os recursos de cada laboratório, para garantir que as amostras sejam obtidas de forma reprodutível, favorecendo a troca de dados entre diferentes grupos de trabalho, sua avaliação e análise de forma ampla. O principal objetivo é acelerar o desenvolvimento de novos métodos diagnósticos e a identificação de biomarcadores para a doença de Chagas.

**Resumo do autor**

O diagnóstico da doença de Chagas requer equipamentos caros e pessoal capacitado, cuja falta dificulta o acesso ao diagnóstico e tratamento em vastas áreas de regiões endêmicas. Além disso, a avaliação da eficácia do tratamento é complicada devido à lenta a reversão da reatividade sorologia anti-*T. cruzi*. Portanto, há uma necessidade urgente de biomarcadores de eficácia terapêutica precoce e prognóstico da doença, bem como ferramentas mais práticas de diagnóstico. Para realizar estudos que abordem essas necessidades, é fundamental ter coleções de amostras clínicas de boa qualidade, rastreabilidade e informações clínico-epidemiológicas associadas de forma adequada. Neste trabalho, fornecemos um protocolo padrão para coletar, processar, armazenar e transportar amostras clínicas de pacientes com doença de Chagas. O manual foi elaborado após alcançar um consenso entre os especialistas da Rede NHEPACHA, uma coalizão de pesquisadores clínicos e acadêmicos das Américas e Espanha que busca a identificação e validação de novos biomarcadores e diagnósticos para a doença de Chagas.

# **Introdução**

A doença de Chagas, causada pelo protozoário parasita *Trypanosoma cruzi* (*T. cruzi*), afeta mais de 6 milhões de pessoas em todo o mundo e a zoonose parasitária mais importante da América Latina (1). Além disso, devido aos importantes fluxos migratórios, tornou-se um problema global nas últimas décadas, com casos diagnosticados regularmente na Europa, América do Norte, Oceania e Ásia (2).

A doença apresenta duas fases: aguda e crônica. A primeira, que dura de quatro a oito semanas, é em sua maioria assintomática, mas pode ser letal em 5% dos casos, especialmente em crianças e pacientes imunossuprimidos. A maioria dos indivíduos infectados progride para infecção crônica (3), que pode durar décadas, durante as quais a presença do parasita persiste sem causar sintomatologia clínica. No entanto, com o tempo, aproximadamente 30% dos indivíduos cronicamente infectados desenvolverão as manifestações cardíacas e digestivas características da doença (3).

Há quase sessenta anos, existem dois medicamentos disponíveis: benznidazol e nifurtimox. Ambos são altamente eficazes na fase aguda, mas menos eficazes na fase crônica, quando a maioria dos casos é diagnosticada. Além disso, ambas requerem esquemas de tratamento longos, frequentemente associados a efeitos adversos que podem levar à interrupção do tratamento. O diagnóstico precoce é crucial para melhorar a eficácia do tratamento, no entanto, os sintomas na fase aguda são frequentemente inespecíficos, dificultando sua detecção oportuna (1).

O diagnóstico da fase crônica da infecção é indireto e depende da detecção de anticorpos anti- *T. cruzi* específicos. Dada a notável diversidade antigênica do parasito, para confirmar a infecção, a Organização Mundial da Saúde (OMS) e a Organização Pan-Americana da Saúde (OPAS) recomendam o uso de dois testes sorológicos detectando conjuntos antigênicos diferentes, a fim de alcançar um diagnóstico confirmado da infecção. As técnicas sorológicas apresentam alguns inconvenientes, como a possível reação cruzada com imunoglobulinas contra outros parasitas intimamente relacionados, como *Leishmania* spp., ou a impossibilidade de avaliar a eficácia do tratamento, devido ao longo período necessário para que os títulos dos anticorpos voltem aos níveis normais após o tratamento bem-sucedido (4). Isso também é problemático no contexto de ensaios clínicos que avaliam novos medicamentos contra a doença. Também, as técnicas sorológicas requerem pessoal treinado e equipamentos relativamente caros para serem realizados, inviabilizando seu uso regular na maioria das unidades básicas de saúde de regiões endêmicas.

Em resposta a este problema, métodos imunocromatográficos baratos, conhecidos como testes de diagnóstico rápido (RDTs) foram desenvolvidos recentemente (5,6). Esses são fáceis de usar, não requerem eletricidade ou controle de temperatura e podem fornecer um resultado em menos de uma hora.

Embora os RDTs sejam usados para triagem em países como Bolívia, Colômbia ou Paraguai, os métodos sorológicos convencionais ainda são necessários para confirmar o diagnóstico (6). Uma alternativa que foi proposta é o uso combinado de dois (ou três no caso de discordância) RDTs com diferentes alvos antigênicos (7−11). Embora os RDTs tenham mostrado alta sensibilidade e especificidade em algumas regiões com alta prevalência de infecção (como certas áreas da Bolívia, norte da Argentina e na Colômbia), seu desempenho é questionado em outras regiões geográficas, onde diferentes cepas parasitas circulantes podem predominar, assim como em áreas de baixa prevalência de infecção (6,12). Portanto, o desenvolvimento de um RDTs “universal” ou RDTs adaptados regionalmente com ampla cobertura geográfica representa um objetivo extremamente importante.

Por outro lado, a falta de biomarcadores para a avaliação do prognóstico da doença e a avaliação da eficácia do tratamento na doença de Chagas representam um desafio significativo para pacientes, prestadores de cuidados médicos e pesquisadores clínicos. Esses biomarcadores poderiam identificar aqueles com maior risco de danos aos órgãos e facilitar o desenvolvimento de novos testes para avaliar a eficácia terapêutica dos medicamentos atualmente disponíveis ou em teste clínico (12−14).

Identificar novos biomarcadores de resposta ao tratamento e de progressão representaria um grande avanço no campo. Diversas moléculas candidatas foram avaliadas, mas a maioria dos estudos incluiu apenas um limitado número de amostras clínicas, de pacientes com curtos períodos de acompanhamento pós-tratamento (12). Quanto aos testes diagnósticos, coortes maiores, bem classificadas e caracterizadas de diversas origens geográficas são necessárias para resolver essa questão. As coortes devem incluir controles não infectados e indivíduos com conversão sorológica após períodos de monitoramento prolongados para serem classificados com segurança como controles positivos de cura.

Avaliar marcadores de resposta terapêutica precoce requer um acompanhamento prolongado dos participantes, idealmente abrangendo cinco anos ou mais após o tratamento, para estudar a progressão crônica da doença (13). Para que a análise das amostras, seguindo metodologias similares, seja comparável em diferentes regiões/países, elas sempre devem ser obtidas, processadas e armazenadas seguindo protocolos padronizados e reprodutíveis, e devem estar vinculadas a dados clínico-epidemiológicos de qualidade.

Amostras biológicas podem ser armazenadas em biobancos e biorrepositórios de pesquisa*.* Embora não haja um conceito universalmente aceito para um biobanco, ele pode ser definido como uma instalação pública ou privada sem fins lucrativos, onde amostras biológicas são armazenadas e anotadas com metadados relevantes. Essas amostras podem ser usadas por terceiros em pesquisas ou projetos de saúde. Já os biobancos são tipicamente entendidos como espaços centralizados que recebem amostras de diferentes biorrepositórios. Ao contrário dos biobancos, os biorrepositórios geralmente pertencem a grupos de pesquisa individuais e não são compartilhados regularmente com outros grupos.

Neste manual, descrevemos os diferentes aspectos a serem considerados ao gerar e manter uma coleção de amostras clínicas obtidas de pacientes com doença de Chagas. Informações detalhadas foram preparadas de acordo com o BRISQ (Biospecimen Reporting from Improved Study Quality, (15)). A metodologia descrita é resultado de uma colaboração da iniciativa da pesquisa NHEPACHA (a sigla para a iniciativa “Novas Ferramentas para o Diagnóstico e Avaliação de Pacientes com doença de Chagas”, lendo em espanhol).

O NHEPACHA foi criada em 2011, com o objetivo de identificar e validar o uso de novos biomarcadores para a doença de Chagas. Atualmente é formado por 18 grupos de pesquisa de 9 países. A estrutura descrita neste documento foi revisada e aprovada pela comissão de especialistas da rede. O objetivo final é facilitar a criação de novas coleções de amostras clínicas obtidas de pacientes com doença de Chagas, bem como aprimorar as já existentes, para que possam ser utilizadas em estudos multicêntricos internacionais para avaliação de novas alternativas terapêuticas e a identificação de biomarcadores de eficácia terapêutica e progressão do paciente.

# **2. Ética**

A extração, processamento e utilização das amostras devem ser realizadas em total conformidade com a revisão atual da Declaração de Helsinque (64ª Assembleia Geral, Fortaleza, Brasil, 2013), bem como em conformidade com a regulamentação local respectiva do país onde as amostras estarão recolhidas. Além disso, aderir à legislação europeia, conhecida por suas rigorosas medidas de proteção de dados, poderia facilitar a transferência de amostras para futuros projetos de pesquisa.

As amostras devem ser obtidas apenas após o participante assinar um formulário de consentimento informado (TCLE). O TCLE deve incluir o objetivo/s da pesquisa e o uso pretendido das amostras (atuais e futuras, se necessário). Tanto o TCLE quanto o protocolo do estudo correspondente devem ter sido aprovados por um comitê de ética em pesquisa clínica independente. Este comitê confirmará que o projeto está em conformidade com a regulamentações legais e éticas existentes, enquanto os pesquisadores são responsáveis pela rastreabilidade das amostras e pela confidencialidade de quaisquer dados derivados.

O material biológico, bem como os dados clínicos a ele associados, devem ser incluídos na coleta e disponibilizados a qualquer grupo de pesquisa que os solicite, após a obtenção da correspondente aprovação ética. As amostras biológicas e seus metadados podem ser armazenados pelo período de tempo descrito no protocolo de estudo específico e formulários do TCLE. Tanto as amostras quanto seus metadados devem sempre ser codificados ou anonimizados, e apenas pessoal autorizado deve ser capaz de vincular esses códigos à identidade do participante. Preferencialmente, os dados clínicos e quaisquer outras informações relevantes devem ser armazenados em registros físicos e eletrônicos, gerenciados pelo centro de estudo responsável. Esta informação só pode ser trabalhada e compartilhada no contexto de um projeto de pesquisa biomédica em andamento e de acordo com seus protocolos aprovados e formulários do TCLE.

O uso retrospectivo de amostras para fins não divulgados no momento da coleta deve estar de acordo com as informações incluídas nos formulários de TCLE. Caso contrário, outro TCLE assinado pelo participante deve ser obtido. Por outro lado, solicitar amostras biológicas que já foram coletadas, e seus metadados, apresenta vários desafios que devem ser considerados: procedimentos e diretrizes rigorosos, abrangendo permissões, documentação e conformidade com padrões éticos e regulatórios que podem variar entre países.

# **3. Extração de amostras**

Os tipos de amostras extraídas e seu processamento (Seção 4: Processamento de amostras) serão determinados com base na natureza dos ensaios em que serão utilizadas. Se possível, os participantes devem ser recrutados assim que o diagnóstico da infecção por *T. cruzi* for confirmado, tentando garantir sua assistência para visitas de acompanhamento. Dependendo do tipo de estudo planejado, os participantes que iniciaram o tratamento antiparasitário podem ser excluídos, independentemente de terem sido concluídos ou não. Este seria o caso, por exemplo, de estudos avaliando o desempenho de novos diagnósticos sorológicos (por exemplo, RDTs). É improvável que o estágio de infecção de um indivíduo seja identificado durante a primeira visita. Mesmo após as primeiras amostras serem inicialmente coletadas, um diagnóstico pode levar várias semanas, principalmente em áreas endêmicas. Além disso, o preenchimento do formulário de dados clínicos exigirá uma série de exames (por exemplo, eletrocardiografia), e essas informações precisaram estar vinculadas à amostra coletada.

A extração de as amostras deve ser sempre realizada em centro de saúde com infraestrutura adequada. O tempo entre a coleta da amostra e as próximas consultas de acompanhamento dependerá do diagnóstico obtido na primeira consulta. Normalmente, e de acordo com as recomendações clínicas vigentes, amostras de pessoas infectadas que cumpram os critérios para receber tratamento antiparasitário devem ser obtidas imediatamente antes e após o término do esquema de tratamento, bem como seis meses após o término e uma vez por ano a partir desse ponto em diante. Amostras de controles não infectados devem, preferencialmente, ser obtidas um, cinco e dez anos após a visita de recrutamento do estudo. Em todos os casos, as amostras devem ser corretamente etiquetadas com a codificação correspondente a cada paciente, a data da extração e o tipo de amostra obtida. A codificação das amostras estará associada à do laboratório responsável pelo processamento. Deveria ser permitida uma certa flexibilidade na janela de visitas (por exemplo, 2-3 meses antes ou depois da data agendada), devido aos desafios de manter-se na agenda.

Depois de coletadas, as amostras devem ser idealmente transportadas para o laboratório de referência a 4ºC e armazenadas nessa temperatura até o processamento, que deve ser feito dentro de um período não superior a 24 horas após a extração (Seção 4: Processamento da amostra). A Figura 1 representa a coleta da amostra, transporte, armazenamento e processamento da amostra.

**Figura 1. Algoritmo de coleta, transporte, armazenamento e processamento da amostra.**


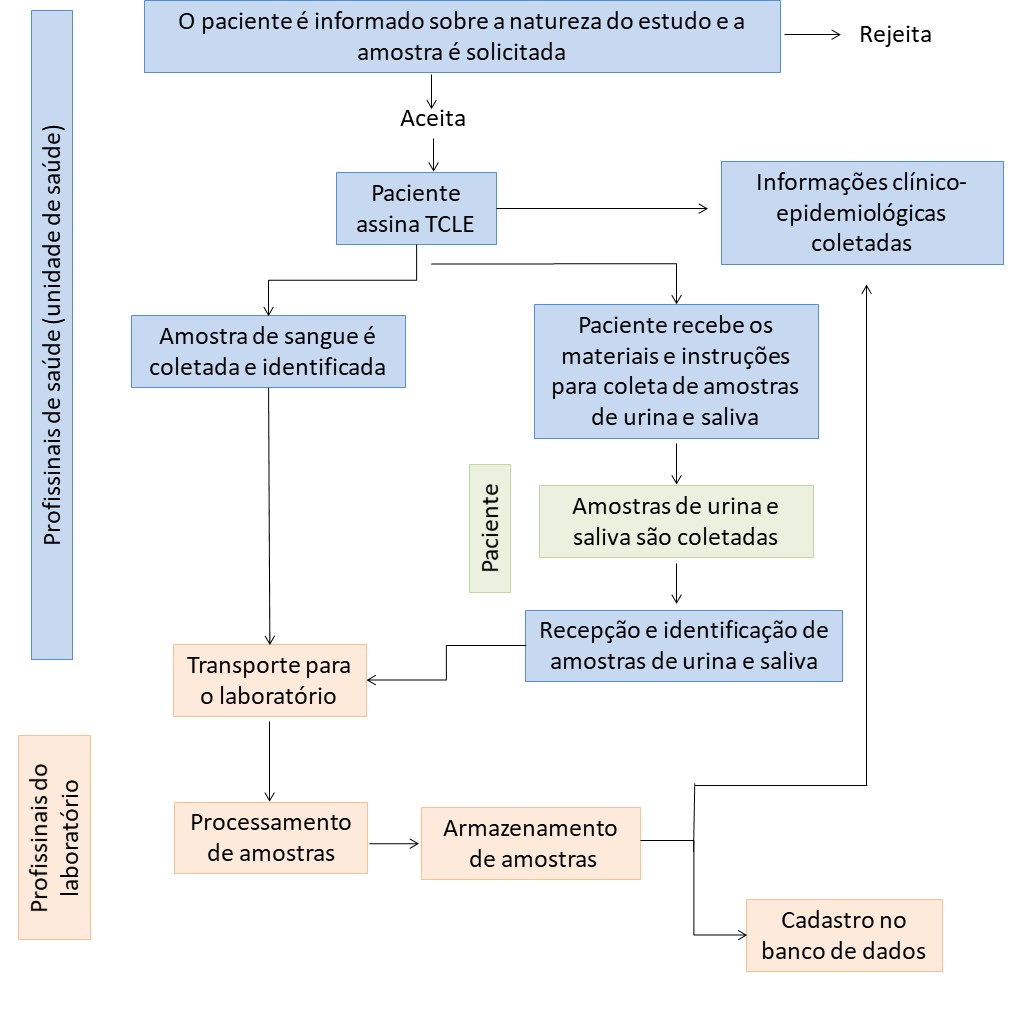


## **3.1 Extração de sangue**

### **3.1.1 Procedimentos gerais para extração de sangue**

A extração de sangue deve ser realizada por pessoal treinado para reduzir o desconforto do participante, sem comprometer a quantidade e a qualidade da amostra.

O sangue é geralmente obtido por punção venosa utilizando agulha e seringa ou um sistema de extração a vácuo de plástico intercambiável (16). A extrações de sangue venoso pode ser realizada seguindo qualquer técnica aplicada regularmente no centro de saúde. No entanto, algumas recomendações gerais devem ser seguidas para reduzir o impacto da extração nos processos analíticos subsequentes. Por exemplo, deve-se deixar o sujeito sentar ou deitar, desinfetar o local da punção com álcool isopropílico 70% e usar torniquete e pressionar a ferida da punção por pelo menos dois minutos para favorecer a hemostasia (Arquivo S7).

Para a construção de biblioteca de amostras, será necessário extrair pelo menos duas amostras: uma em um tubo tratado com anticoagulante e outra em um tubo não tratado (Figura 2). Para fins de generalização dos resultados e com base na disponibilidade de tubos com anticoagulante, o ácido etilenodiaminotetracético (EDTA, geralmente EDTA-K2 por ser o mais comum) deve ser preferencialmente utilizado como anticoagulante de escolha, se possível.

### **3.1.2 Sangue total e suas frações**

Três amostras diferentes são obtidas de uma extração de sangue: soro, plasma e sangue total. Essas amostras devem ser coletadas em tubos individuais e extraídas em ordem sequencial, começando pelo tubo não tratado destinado ao soro, seguindo-se os tubos tratados com EDTA para plasma e sangue total. Isso é importante para evitar a contaminação cruzada de amostras com EDTA e aditivos semelhantes.

1. **O soro** é a amostra mais utilizada em estudos de busca de novos biomarcadores e para avaliação de novas técnicas diagnósticas. Deve ser coletado em tubos não tratados (secos). Se possível, devem ser utilizados tubos contendo um gel inerte para favorecer a separação do soro, pois evitam a contaminação do soro com outros hemocomponentes.
2. **Sangue total** é útil para a avaliação dos RDTs, bem como para a realização de técnicas parasitológicas, como o micrométodo (4). É também a amostra mais comumente utilizada no diagnóstico molecular da infecção, seja por reação em cadeia da polimerase (PCR qualitativo e quantitativo) ou LAMP (17,18). Esta amostra é tipicamente coletada em tubos tratados com EDTA, que devem ser conservados preferencialmente a -80 ou -20 ºC). Caso as amostras precisem ser mantidas a 4ºC por longos períodos de tempo antes do diagnóstico molecular, elas devem ser tratadas com cloridrato de guanidina 6 M – EDTA 0.2 M (GE), pH 8.00 na proporção de 1:1 (vol.:vol.). A GE é excelente para preservar amostras para diagnóstico molecular, mesmo que sejam armazenadas em temperatura ambiente, algo particularmente útil em áreas endêmicas. Tubos pré-carregados com GE também podem ser usados para processar várias amostras ao mesmo tempo. No entanto, a guanidina é um reagente caro, o que pode limitar sua ampla utilização em ambientes endêmicos. Um protocolo para a preparação deste reagente é detalhado no Arquivo S8.

Se as amostras forem usadas para à avaliação de testes moleculares LAMP baseados na reatividade do substrato de calceína (por exemplo, o *T. cruzi*-LAMP desenvolvido pela Eiken Chemical Co., Ltd, Tóquio, Japão), é aconselhável evitar o uso de EDTA, pois isso pode afetar o desempenho do ensaio (18). O uso de tubos tratados com heparina, em vez disso, representa uma alternativa nesses casos. Este também é o anticoagulante preferido para usar em amostras que serão analisadas pelo micrométodo, um teste parasitológico direto baseado em microscopia. Enquanto o LAMP e o PCR têm sensibilidade e especificidade quase idênticas (19), o primeiro tem a vantagem de exigir uma infraestrutura mais simples, facilitando seu uso em áreas endêmicas, principalmente para o diagnóstico da doença de Chagas congênita.

1. **O plasma** é obtido a partir de amostras coletadas em tubos tratados com anticoagulante, preferencialmente com EDTA, por ser este o composto mais comumente utilizado para a coleta de amostras de sangue total, facilitando os aspectos logísticos do processo de coleta de amostras. Outros anticoagulantes com diferentes mecanismos de ação podem comprometer a qualidade da amostra.

Se necessário, recomenda-se priorizar a obtenção de soro em detrimento do plasma, pois é mais fácil de obter e tem melhor desempenho em diferentes sistemas de diagnóstico. No entanto, é importante considerar que os fatores de coagulação não estão presentes no soro; e estes podem ser úteis para a identificação de biomarcadores envolvidos na ativação da via de coagulação (20). Amostras de sangue total devem ser obtidas sempre que possível, para diagnóstico molecular.

### **3.1.3 Volume de sangue extraído**

O volume total de sangue extraído dependerá da idade do participante. O volume total ideal extraído em participantes com 18 anos ou mais não deve exceder 10 mL em tubos tratados com EDTA (desejável 5 mL em cada um dos dois tubos) e 5 mL no tubo não tratado 🡪15 mL do volume total de sangue extraído. Para participantes entre 5 e 18 anos, o volume total extraído nos tubos tratados com EDTA deve ser de 5 mL (2,5 mL em cada tubo) e de 5 mL nos tubos não tratados para o soro 🡪 10 mL do volume total de sangue extraído. Em crianças de 2 a 5 anos de idade, o volume mínimo possível de sangue deve ser extraído, de acordo com as diretrizes pediátricas locais (21,22). Neonatos e crianças menores de 2 anos constituem outro grupo de interesse, principalmente para o estudo sorológico dentro do algoritmo de diagnóstico da doença de Chagas congênita. O sangue dos neonatos pode ser obtido por meio de extração venosa na parte de trás da mão ao nascer ou através da coleta de gotas de sangue do calcanhar (aproximadamente 500 µL). A partir dos nove meses de idade, seria possível obter cerca de 1 mL de sangue por punção venosa no braço.

Se possível, uma fração do sangue coletado (250 - 500 µl) deve ser transferida para tubos tratados com heparina, para poder realizar os ensaios LAMP. Esses ensaios também podem ser realizados usando manchas de sangue seco (DBS) coletadas diretamente em papel de filtro (por exemplo cartões FTA (QIAcard Flinders Technology Associates (FTA®)™ Classic cards (Qiagen, UK)) (23).

## **3.2 Coleta de urina e saliva**

O uso de técnicas sorológicas para o diagnóstico da infecção pelo *T. cruzi* em amostras de urina (24) ou saliva (25) representa uma alternativa promissora e não invasiva aos métodos existentes. Portanto, a inclusão dessas amostras em coleções clínicas poderia ser considerada. No entanto, a utilidade da urina e da saliva é relativamente restrita em comparação com o sangue quando a priorização é necessária, especialmente ao considerar o espaço disponível e os custos associados à manutenção do armazenamento das amostras. A coleta de urina é realizada diretamente pelos participantes, após instruções verbais e escritas claras (Arquivo S7). Embora esta amostra possa ser recolhida no domicílio, é aconselhável fazê-la no centro de saúde onde serão recolhidos os outros tipos de amostras, durante a mesma visita. Preferencialmente, 5-10 mL da primeira urina da manhã devem ser coletados (26).

A coleta de amostras de saliva também é realizada diretamente pelo participante (Arquivo S7), preferencialmente no centro de saúde, assim como para o restante das amostras. O participante deve abster-se de beber qualquer coisa que não seja água, comer, fumar, escovar os dentes ou mascar chiclete por pelo menos uma hora antes da coleta da amostra. Preferencialmente, 5-10 mL de saliva devem ser coletados.

# **4. Processamento de amostras**

Todas as amostras humanas devem ser consideradas potencialmente perigosas devido ao seu risco biológico intrínseco e, portanto, devem ser processadas em capela de fluxo laminar tipo II (BSL-2). Isso protegerá o operador e minimizará o risco de contaminação das amostras. Todos os tubos devem ser rotulados antes do processamento das amostras, indicando o código de identificação do participante, o tipo de amostra e a data do processamento.

Embora exija mais espaço de armazenamento, é aconselhável preparar 2-3 alíquotas de todas as amostras processadas. Por exemplo, preferencialmente, três alíquotas em criotubos O-ring com tampa de rosca devem ser utilizados para armazenamento a longo prazo. A produção de apenas duas alíquotas é aceitável, se o volume total da amostra for limitado. No caso de acesso limitado às instalações de armazenamento, é possível preparar uma única alíquota e posteriormente subaliquotar tal amostra após ser descongelada pela primeira vez. O número de vezes que uma amostra foi descongelada deve ser anotado, pois ciclos subsequentes de congelamento/descongelamento podem comprometer a qualidade da amostra. Se a amostra foi preparada diluindo-a em igual volume de glicerol (previamente autoclavado e com pelo menos 99% de pureza), ela pode ser descongelada até dez vezes. Amostras sem glicerol não devem ser descongeladas mais de três vezes. Um procedimento operacional padrão para o processamento de amostras é apresentado no Arquivo S8.

## **4.1 Soro**

Para a separação do soro, o tubo não tratado deve ser centrifugado a 1600 g por dez minutos, em temperatura ambiente. Na ausência de uma centrífuga, é possível que um coágulo se forme e precipite espontaneamente, embora isso deva ser evitado, quando possível. Recomenda-se pipetar 2-3 mL da amostra e misturá-la com igual volume de glicerol, antes de aliquotar o volume final em criotubos com tampa de rosca. Esse processo é importante ser realizado em locais onde o acesso à energia elétrica é irregular, pois a queda de luz pode comprometer a integridade das amostras armazenadas. O uso de glicerol impedirá o uso das amostras para avaliação do desempenho do RDT ou estudos de produção de citocinas. Se esses estudos forem realizados, alíquotas extras sem glicerol devem ser preparadas.

## **4.2 Sangue total com guanidina**

Para o processamento de sangue total, o volume total (cerca de 5 mL) de um dos tubos tratados com EDTA deve ser misturado com um volume igual de GE (ref G3272, Sigma-Aldrich). Isso geralmente deve ser feito em um tubo Falcon de 15 mL e o volume total obtido geralmente fica em torno de 10 mL. Depois de misturar até homogeneizar a solução por inversão, dois criotubos com tampa de rosca de 5 mL devem ser criados e armazenados para evitar derramamentos. A guanidina é um agente caotrópico que lisa os eritrócitos, preservando a integridade dos ácidos nucléicos.

## **4.3 Plasma**

Para a obtenção do plasma, o outro tubo tratado com EDTA deve ser centrifugado a 1200 g por dez minutos em temperatura ambiente. Um volume de pelo menos 2 mL deve ser obtido após a centrifugação. Quanto ao soro, o volume de plasma resultante pode ser misturado com uma quantidade igual de glicerol. Conforme discutido anteriormente, a produção de alíquotas extras livres de glicerol pode ser necessária dependendo do objetivo com o qual as amostras foram coletadas. Em ambos os casos, recomenda-se preparar alíquotas de 1 - 2 mL.

## **4.4 Saliva**

As amostras de saliva devem ser centrifugadas a 1000g por 5 minutos em temperatura ambiente para precipitar o muco. O sobrenadante restante deve ser coletado em criotubos de rosca (1 - 2 mL por tubo).

## **4.5 Urina**

A urina não precisa ser centrifugada. No entanto, é importante observar quaisquer alterações observadas na amostra (por exemplo: aumento da turbidez ou presença de sangue), pois podem afetar a interpretação dos resultados. Essas amostras também devem ser armazenadas em criotubos de rosca de 1 - 2 mL.

**Figura 2: Resumo das amostras coletadas rotineiramente em um laboratório clínico de doença de Chagas.** Número sugerido de alíquotas e volumes a serem coletados são mostrados (Figura criada com Biorender , https://biorender.com/ )


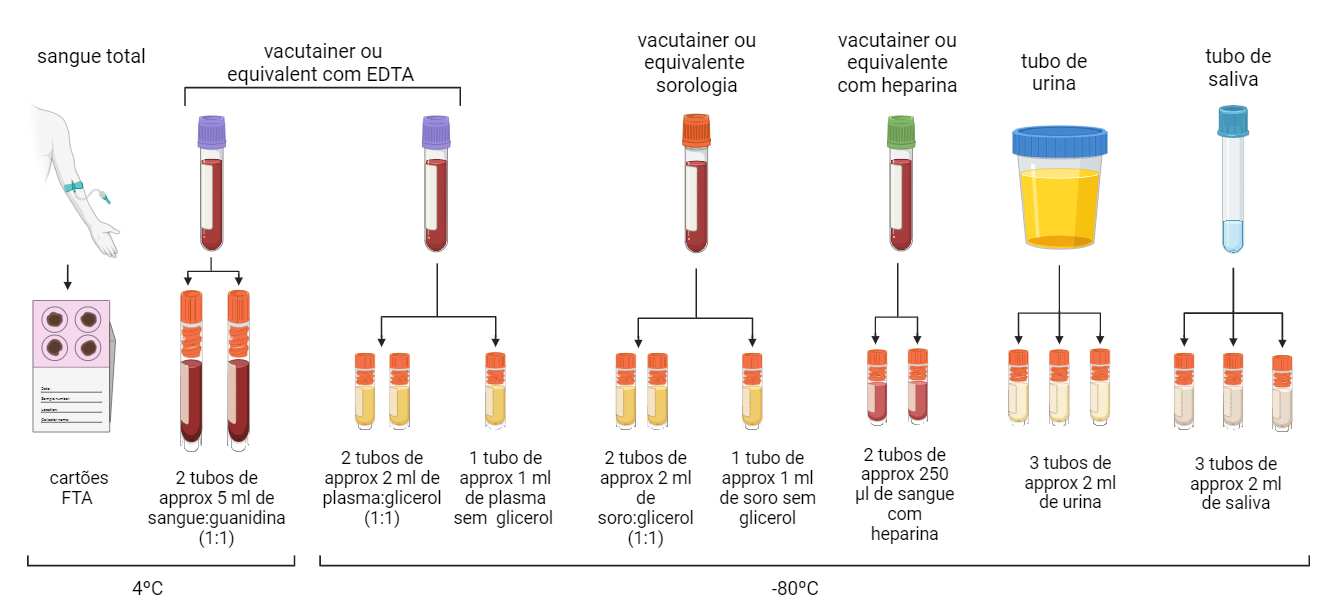


# **5. Armazenamento de amostras**

Depois de processadas e devidamente rotuladas (uma folha de rotulagem está disponível no Arquivo S8), é necessário registrar o local e as condições de armazenamento de cada amostra. Esse registro também deve incluir o número de vezes que uma determinada amostra foi descongelada, juntamente com as respectivas datas e finalidade. O número de ciclos de descongelamento também deve ser anotado diretamente no tubo. A Tabela 1 resume as condições ideais e aceitáveis nas quais diferentes amostras podem ser armazenadas. A maioria das amostras pode ser armazenada com segurança a -70ºC sem comprometer sua qualidade, e a temperatura pode, de fato, contribuir para os protocolos de economia de energia do laboratório. Para amostras destinadas a estudos de produção de citocinas devem ser sempre armazenadas a -70ºC ou menos.

Em regiões endêmicas, onde armazenar as amostras a -80ºC é logisticamente difícil, ou onde o acesso à eletricidade é irregular, amostras de soro e plasma podem ser misturadas com azida sódica e armazenados a 4ºC por longos períodos de tempo sem comprometer sua qualidade. No entanto, isso é aceitável apenas para amostras a serem usadas em testes diagnósticos sorológicos e também pode ser feito para reduzir o número de ciclos de descongelamento aos quais os controles usados nesses testes são expostos. No entanto, este método não deve ser usado para armazenar amostras destinadas a estudos de identificação de biomarcadores.

**Tabela 1. Temperaturas recomendadas para o armazenamento a longo prazo de amostras clínicas**

| **Amostra** | **Temperatura ideal (ºC)** | **Temperatura aceitável (ºC)** |
| --- | --- | --- |
| **Cartões de sangue total com guanidina ou FTA** | 4 | Temperatura ambiente |
| **Sangue total-EDTA/heparina** | -80 | -20 |
| **Sérum** | -80 | -20 |
| **Plasma** | -80 | -20 |
| **Saliva** | -80 | -20 |
| **Urina** | -80 | -20 |

# **6. Gestão de dados e anotação da informação clínica e laboratorial**

Para ser realmente útil em pesquisa, qualquer coleta de amostras clínicas deve ser devidamente anotada com seus dados clínico-epidemiológicos associados. Da mesma forma, os TCLE usados para obter as amostras também devem ser armazenados. É fundamental registrar imediatamente qualquer alteração na coleção (incluindo o uso total ou parcial e descarte de amostras). Diante dessas entradas de informações relacionadas, mas distintas, é altamente recomendável manter todos os registros em um conjunto de dados centralizado, cujo nível de sofisticação dependerá dos recursos disponíveis de cada laboratório e do número de amostras manipuladas, desde registros em papel até conjuntos de dados informáticos multiusuários em tempo real. Esses conjuntos de dados devem incluir a localização exata de cada amostra e qualquer informação clínica e epidemiológica relevante. Para facilitar o processo de coleta de dados, é aconselhável preparar questionários para a captura, validação, armazenamento e gestão dos metadados resultantes. Embora esses questionários possam ser impressos, é altamente recomendável armazenar também uma cópia digital de cada um deles. Nos questionários (como aquele elaborado por González e colaboradores na Rede NHEPACHA, atualmente submetido para revisão), são registradas informações clínicas e epidemiológicas, bem como informações laboratoriais correspondentes a cada tipo de amostra, volume, data de coleta e processamento, juntamente com qualquer incidente possível relacionado a ela.

# **7. Transporte de amostras**

Antes que qualquer amostra possa ser transferida de um centro para outro, um acordo deve ser assinado entre ambas as instituições e todos os requisitos exigidos por lei devem ser cumpridos. Tipicamente, um contrato de transferência de material (MTA) entre os dois centros deve ser arranjado antecipadamente, e uma lista das amostras transportadas juntamente com as licenças de importação necessárias e uma fatura alfandegária.

Preenchidas todas as documentações exigidas, as amostras devem ser acondicionadas de acordo com a legislação vigente, e de acordo com a classificação de risco biológico da amostra, que deverá ser declarada pela instituição expedidora das amostras, seguindo as orientações da respectiva autoridade de transporte.

Para garantir a qualidade das amostras postadas, a temperatura de transporte deve ser registrada e mantida constante. Para isso, é recomendável incluir um registrador de dados de temperatura na embalagem, se possível. Preferencialmente, as amostras de soro, plasma, urina e saliva devem ser transportadas congeladas em gelo seco à temperatura constante de -70ºC. É importante considerar que o gelo seco é classificado como material perigoso e deve ser rotulado adequadamente (26). Senão for possível garantir esta temperatura, as amostras podem ser transportadas a -20ºC, ou utilizando embalagens de gel refrigerante. Em alguns casos, o transporte à temperatura ambiente pode ser aceitável, especialmente se as amostras tiverem sido misturadas com glicerol, embora seja importante observar que elas teriam sido descongeladas uma vez antes do envio.

Em todos os casos, deve-se utilizar uma embalagem grande o suficiente para conter as amostras e o método de refrigeração escolhido. É aconselhável colocar as amostras entre duas camadas do agente refrigerante, para garantir a estabilidade de sua temperatura.

A instituição receptora é responsável por verificar o conteúdo do pacote no momento da chegada e comunicar qualquer incidência detectada ao remetente o mais rápido possível.

# **8. Conclusão**

Os procedimentos descritos neste manual destinam-se a orientar o estabelecimento padronizado de novos repositórios de amostras clínicas para o estudo da doença de Chagas. Amostras rastreáveis e de alta qualidade são essenciais para a identificação de novos biomarcadores para o diagnóstico e prognóstico da doença de Chagas e para monitorar a resposta dos pacientes ao tratamento. É recomendado utilizar este guia em combinação com o guia do questionário clínico (elaborado por González e colaboradores, submetido para revisão) para cada amostra.

**Agradecimentos**

Gostaríamos de agradecer à DND*i* e Fundação Mundo Sano por seu contínuo envolvimento em apoiar a Rede NHEPACHA. MCT e MCL foram apoiados pela Bolsa PID2019-109090RB-100/AEI/10.13039/501100011033 do Programa Estatal I+D+I, Ministério da Ciência e Inovação da Espanha (MICINN). Reconhecemos o apoio da bolsa CEX2018-000806-S financiada por MCIN/AEI/10.13039/501100011033. ILG, JCGF, NMP, JG e JAP reconhecem o apoio da Generalitat de Catalunya por meio do Programa CERCA. JCGF recebeu apoio por meio de uma bolsa da Fundação "la Caixa" (ID 100010434, código da bolsa: LCF/BQ/DI21/11860037).

Grupo de estudo da Rede NHEPACHA:

Janine Ramsey, Angelica Pech May, Alba Valdez Tah, Gilberto Sanchez Gonzalez, Adriana Gonzalez Martinez, Eduardo Ortiz Panozo, Mario J. Grijalva, Jaime A. Costales, Cesar A. Yumiseva, Carolina Herrera, Eileen Velez, Maria de Lourdes Torres, Maria-Jesus Pinazo, Sergio Sosa Estani, Colin Forsyth, Eric Chatelain, Ivan Scandale, Fabiana Barreira, Tayná Marques, Marina Certo, Alejandro Hasslocher, Roberto Saraiva, Mauro Mediano, Andrea Silvestre, Sergio Xavier, Luiz Sangenis, Fernanda Mendes, Gilberto Sperandio da Silva, Andrea Costa, Henrique Veloso, Marcelo Holanda, Flavia Mazzoli, Paula Simplício da Silva, Tania Araujo, Mariana Wagabi, Luciana Garzoni, Constança Brito, Roberto Ferreira, Rita Machado, Raquel Aguiar, Marcelo Abril, Soledad Beron, Alejandro Schijman, Silvia Longhi, Arturo Muñoz-Calderón, Belkisyole Alarcon de Noya, Oscar Noya Gonzalez, Arturo Muñoz, Cecilia Colmenares, Ivan Mendoza, Zoraida Diaz, Raiza Ruiz, Ana Andreina Alviares, María Carmen Thomas, Manuel Carlos Lopez, Adriana Egui, Celia Benitez, Inmaculada Gómez, Francisco Macias Huete, Andres Mariano Ruiz, Rocio Rivero, Mónica Esteva, Margarita Bisio, Marisa Fernandez, Yolanda Hernandez, Julio Alonso Padilla, Joaquim Gascon, Irene Losada Galván, Nieves Martinez Peinado, Juan Carlos Gabaldon, María Gabriela Alvarez, Lococo Bruno, Laucella Susana, Flavio Andrés Tóman Conte, Dr. Enrique Morral, Maria Cecilia Albareda, Fernán Agüero, Emir Salas Sarduy, Alejandro Ricci, Leonel Bracco, Mercedes Didier Garnham, Alejandro Luquetti, Igor Almeida, Ester Sabino, Felipe Guhl, Faustino Torrico

# **Referências**

1. WHO. Chagas disease (American trypanosomiasis). [Cited 31 Jul 2023]. Available from: https://www.who.int/en/news-room/fact-sheets/detail/chagas-disease-(american-trypanosomiasis).

2. Alonso-Padilla J, Cortés-Serra N, Pinazo MJ, Elena M, Abril M, Barreira F, et al. Strategies to enhance access to diagnosis and treatment for Chagas disease patients in Latin America. Expert Rev Anti Infect Ther. 2019;17:145–57. doi: 10.1080/14787210.2019.1577731

3. Pinazo MJ, Gascon J. Chagas disease: from Latin America to the world. Reports Parasitol. 2015;4:7–14.

4. Gállego M, Schijman AG, Alonso-Padilla J. Diagnosis of Trypanosoma cruzi infection: challenges on laboratory tests development and applications. In: Pinazo MJ, Gascon J., editors. Chagas disease. A neglected tropical disease. Springer Nature; 2020, pp. 75–94.

5. Pinazo MJ, Gascon J, Alonso-Padilla J. How effective are rapid diagnostic tests for Chagas disease? Expert Rev Anti Infect Ther. 2021;19:1489-1494. doi: 10.1080/14787210.2021.1873130

6. Lozano D, Rojas L, Méndez S, Casellas A, Sanz S, Ortiz L, et al. Use of rapid diagnostic tests (RDTs) for conclusive diagnosis of chronic Chagas disease - field implementation in the Bolivian Chaco region. PLoS Negl Trop Dis. 2019;13:e0007877. doi: 10.1371/journal.pntd.0007877

7. Egüez KE, Alonso-Padilla J, Terán C, Chipana Z, García W, Torrico F, et al. Rapid diagnostic tests duo as alternative to conventional serological assays for conclusive Chagas disease diagnosis. PLoS Negl Trop Dis. 2017;11:e0005501. doi: 10.1371/journal.pntd.0005501

8. Mendicino D, Colussi C, Moretti E. Simultaneous use of two rapid diagnostic tests for the diagnosis of Chagas disease. Trop Doct. 2019;49:23-26. doi: 10.1177/0049475518813792

9. Lopez-Albizu C, Danesi E, Piorno P, Fernandez M, García Campos F, Scollo K, Crudo F. Rapid diagnostic tests for Trypanosoma cruzi infection: field evaluation of two registered kits in a region of endemicity and a region of nonendemicity in Argentina. J Clin Microbiol. 2020;58:e01140-20. doi: 10.1128/JCM.01140-20

10. Suescún-Carrero SH, Tadger P, Sandoval Cuellar C, Armadans-Gil L, Ramírez López LX. Rapid diagnostic tests and ELISA for diagnosing chronic Chagas disease: systematic revision and meta-analysis. PLoS Negl Trop Dis. 2022;16:e0010860. doi: 10.1371/journal.pntd.0010860

11. Angheben A, Buonfrate D, Cruciani M, Jackson Y, Alonso-Padilla J, Gascon J, et al. Rapid immunochromatographic tests for the diagnosis of chronic Chagas disease in at-risk populations: a systematic review and meta-analysis. PLoS Negl Trop Dis. 2019;13:e0007271. doi: 10.1371/journal.pntd.0007271

12. Cortes-Serra N, Losada-Galvan I, Pinazo MJ, Fernandez-Becerra C, Gascon J, Alonso-Padilla J. State-of-the-art in host-derived biomarkers of Chagas disease prognosis and early evaluation of anti-Trypanosoma cruzi treatment response. Biochim Biophys Acta Mol Basis Dis. 2020;1866:165758. doi: 10.1016/j.bbadis.2020.165758.

13. Pinazo MJ, Thomas MC, Bua J, Perrone A, Schijman AG, Viotti RJ, et al. Biological markers for evaluating therapeutic efficacy in Chagas disease, a systematic review. Expert Rev Anti Infect Ther. 2014;12:479-96. doi: 10.1586/14787210.2014.899150

14. Mendes VG, Rimolo L, de Lima ACB, Ferreira RR, Oliveira LS, Nisimura LM, Horita SIM, Costa AR, da Silva GMS, Sangenis LHC, Mendes FSNS, Sousa AS, Veloso HH, Holanda MT, Mediano MFF, Waghabi MC, Garzoni LR, Moreira OC, Britto C, Cunha AB, Hasslocher-Moreno AM, Saraiva RM. Biomarkers and echocardiographic predictors of cardiovascular outcome in patients with chronic Chagas disease. J Am Heart Assoc. 2023;12(12):e028810. doi: 10.1161/JAHA.122.028810.

15. Moore HM, Kelly AB, Jewell SD, McShane LM, Clark DP, Greenspan R, Hayes DF, Hainaut P, Kim P, Mansfield EA, Potapova O, Riegman P, Rubinstein Y, Seijo E, Somiari S, Watson P, Weier HU, Zhu C, Vaught J. Biospecimen reporting for improved study quality (BRISQ). Cancer Cytopathol. 2011;119:92-101. doi: 10.1002/cncy.20147

16. Vaught JB, Henderson MK. Biological sample collection, processing, storage and information management. IARC Sci Publ. 2011;23–42.

17. Muñoz-Calderón AA, Besuschio SA, Wong S, Fernández M, García Cáceres LJ, Giorgio P, et al. Loop-mediated isothermal amplification of Trypanosoma cruzi DNA for point-of-care follow-up of anti-parasitic treatment of Chagas disease. Microorganisms. 2022;10:909. doi: 10.3390/microorganisms10050909

18. Polley SD, González IJ, Mohamed D, Daly R, Bowers K, Watson J, et al. Clinical evaluation of a loop-mediated amplification kit for diagnosis of imported malaria. J Infect Dis. 2013;208:637-44. doi: 10.1093/infdis/jit183

19. Besuschio SA, Llano Murcia M, Benatar AF, Monnerat S, Cruz I, Picado A, et al. Analytical sensitivity and specificity of a loop-mediated isothermal amplification (LAMP) kit prototype for detection of Trypanosoma cruzi DNA in human blood samples. PLoS Negl Trop Dis. 2017;11:e0005779. doi: 10.1371/journal.pntd.0005779

20. Pinazo MJ, Posada Ede J, Izquierdo L, Tassies D, Marques AF, de Lazzari E, et al. Altered hypercoagulability factors in patients with chronic Chagas disease: potential biomarkers of therapeutic response. PLoS Negl Trop Dis. 2016;10:e0004269. doi: 10.1371/journal.pntd.0004269

21. Martorell LLCM. Hemocultivos en el Instituto de Hematología e Inmunología: optimizando la toma de muestra. Rev Cuba Hematol Inmunol y Hemoter. 2019;37:1–16.

22. Calvo Cillán A. Utilidad de la extracción de un volumen adecuado de sangre para aumentar la rentabilidad de los hemocultivos en pediatría. M. Sc. Thesis. Universidad Internacional de Andalucía. 2018. Available from: https://dspace.unia.es/bitstream/handle/10334/3889/0857_Calvo.pdf?sequence=1&isAllowed=y

23. Longhi SA, García Casares LJ, Muñoz-Calderón AA, Alonso-Padilla J, Schijman AG. Combination of ultra-rapid DNA purification (PURE) and loop-mediated isothermal amplification (LAMP) for rapid detection of Trypanosoma cruzi DNA in dried blood spots. PLoS Negl Trop Dis. 2023;17(4):e0011290. doi:10.1371/journal.pntd.0011290

24. Castro-Sesquen YE, Gilman RH, Galdos-Cardenas G, Ferrufino L, Sánchez G, Valencia Ayala E, et al. Use of a novel Chagas urine nanoparticle test (chunap) for diagnosis of congenital Chagas disease. PLoS Negl Trop Dis. 2014;8:e3211. doi: 10.1371/journal.pntd.0003211

25. Cortes-Serra N, Pinazo MJ, de la Torre L, Galizzi M, Gascon J, Bustamante JM. Diagnosis of Trypanosoma cruzi infection status using saliva of infected subjects. Am J Trop Med Hyg. 2018;98:464-467. doi: 10.4269/ajtmh.17-0141.

26. Campbell LD, Astrin JJ, DeSouza Y, Giri J, Patel AA, Rawley-Payne M, Rush A, Sieffert N. Best practices: recommendations for repositories. 4st ed. Vancouver: ISBER International Society for Biological and Environmental Repositories; 2018.
