# Supplementary material for: A guide for the generation of repositories of clinical samples for research on Chagas disease
Source: PLoS Negl Trop Dis. 2024 Aug 15;18(8):e0012166. doi: 10.1371/journal.pntd.0012166 (PMC11326570; doi:10.1371/journal.pntd.0012166)
Supplement: S7 File — (DOCX) [file pntd.0012166.s007.docx]

**S7. Procedimento operacional padrão para coleta de amostras clínicas para uso em repositórios de pesquisa em doença de Chagas**

1. **Coleta de sangue**

**Material necessário:**

- Bandeja
- Algodão
- Álcool isopropílico
- Torniquetes/Garrote
- Luvas cirúrgicas
- Agulha e tubos Vacutainer
- Suporte para tubos
- 1 Tubo coletor não tratado.
- 2 tubos de coleta tratados com EDTA-K2.
- Fita cirúrgica hipoalergênica
- Marcador permanente
- Etiquetas adesivas de identificação
- Recipiente para descarte de risco biológico

**Procedimento para a coleta de amostras de sangue venoso**

1. Verifique a identidade do paciente e os pedidos de teste. Lembre-se de coletar primeiro a amostra no tubo não tratado, para evitar a contaminação.
2. Explique claramente o procedimento ao paciente.
3. Prepare o material de extração: torniquete/garrote, algodão, álcool, fita adesiva, agulha vacutainer, tubos e luvas. Todos devem estar na bandeja.
4. Etiquete cada tubo com o código do paciente correspondente, a data e o tipo de amostra que está sendo coletada.
5. Prepare a agulha vacutainer e coloque no paciente o torniquete/garrote com pressão suficiente para ajudar a localizar a veia, sem comprometer o fluxo sanguíneo. A pressão excessiva pode causar hemólise, colapso venoso e dor, além de comprometer a qualidade da amostra.
6. Identifique a veia mais proeminente e facilmente palpável na fossa antecubital, aplique o álcool para desinfetar a área e deixe secar. Perfure a pele com o bisel da agulha voltado para cima e formando um ângulo de aproximadamente 15º com a superfície da pele.
7. Se as amostras estiverem sendo coletadas em cartões FTA, aplique gotas de sangue diretamente sobre eles. Preencha cada círculo impresso no cartão FTA com uma gota de sangue. É importante que cada círculo tenha aproximadamente o mesmo volume de sangue e que a área impressa esteja totalmente preenchida.
8. Armazenar o cartão FTA em bolsa metálica com sílica gel e conservar a 4ºC até o transporte ao laboratório.
9. Pressione a tampa do tubo de vácuo contra a extremidade livre da agulha do vacutainer e deixe-a encher. Uma vez cheio, retire o tubo da agulha vacutainer e repita o procedimento com os tubos restantes. Lembre-se de coletar primeiro a amostra no tubo não tratado.
10. Retire o último tubo da agulha vacutainer, em seguida retire o torniquete/garrote e a agulha do paciente. Pressione o local da punção com um algodão.
11. Descarte a agulha vacutainer no recipiente de risco biológico correspondente.
12. Os tubos tratados com EDTA-K2 devem ser preenchidos até que o vácuo desapareça para manter uma proporção adequada entre a amostra e o anticoagulante. Uma vez cheio, misture imediatamente por meio de inversão, 5 a10 vezes, para evitar a formação de coágulos.
13. Confirme se todas as amostras necessárias foram coletadas antes de permitir que o paciente saia.
14. Armazene todas as amostras coletadas a 4ºC até o transporte para o laboratório. As amostras devem ser processadas dentro de 24 horas após a extração.
15. **Coleta de saliva**

**Material necessário**

- Tubo coletor de saliva.

**Procedimento para a coleta de amostras de saliva**

O paciente deve receber as seguintes instruções verbalmente e por escrito:

1. **Não** coma, masque chiclete, fume ou beba nada além de água durante pelo menos uma hora antes da coleta da amostra. O uso de batom, *lip balms* (bálsamos labiais) ou cremes labiais e inaladores de esteroides também deve ser evitado imediatamente antes da coleta da amostra. Evite qualquer atividade que possa causar sangramento gengival, incluindo escovar os dentes. Não use ácido ascórbico (vitamina C) ou qualquer enxaguante bucal para estimular a salivação antes de coletar a amostra.
2. Esfregue o palato e as bochechas com a língua para estimular a salivação.
3. Cuspa no tubo de coleta até que o nível de saliva (sem bolhas) atinja a linha de 5 ml. Não colete mais de 10 ml. Bata suavemente o tubo contra uma superfície dura para reduzir o volume das bolhas.
4. Feche bem o tubo coletor.
5. **Coleta da urina**

**Material necessário**

- Recipiente de urina.

**Procedimento para coletar amostras de urina**

O paciente deve receber as seguintes instruções verbalmente e por escrito:

1. Lave as mãos com água e sabão.
2. Separe os lábios vaginais com o auxílio dos dedos ou retraia o prepúcio do pênis e comece a urinar. Não colete o primeiro jato de urina.
3. Continue urinando no recipiente de coleta estéril, evitando qualquer contato entre a pele e o jato.
4. Feche bem o recipiente de coleta, sem tocar em seu interior.
