# Supplementary material for: A guide for the generation of repositories of clinical samples for research on Chagas disease
Source: PLoS Negl Trop Dis. 2024 Aug 15;18(8):e0012166. doi: 10.1371/journal.pntd.0012166 (PMC11326570; doi:10.1371/journal.pntd.0012166)
Supplement: S8 File — (DOCX) [file pntd.0012166.s008.docx]

**S8. Procedimento operacional padrão para o processamento de amostras clínicas para uso em repositórios de pesquisa em doença de Chagas**

**Resumo para a coleta de amostras**

|  | **Sangue total (aproximadamente 15 ml)** |
| --- | --- |
| **Condições** | - 10 ml coletados em 2 tubos tratados com EDTA (tampa roxa). - 5 ml coletados em um tubo não tratado, com gel separador e ativador (tampa vermelha). - 500 µl coletados em um tubo tratado com heparina (tampa verde). |
| **Armazenamento das amostras - até a coleta pela equipe do laboratório** | 4ºC |
| **Transporte** | Designe uma pessoa para coletar e transportar as amostras (as amostras devem ser processadas nas primeiras 24 horas após a extração). |

**Notas gerais**

- Todas as amostras devem ser processadas em cabine segurança biológica classe 2.
- Etiquetas aderentes individuais devem ser confeccionadas para a identificação dos tubos antes do processamento das amostras. Uma vez aplicadas em cada tubo, as etiquetas devem ser cobertas com fita plástica transparente, para evitar seu desgaste.
- Depois de processar as amostras, os dados no repositório deve ser imediatamente atualizado.

**Reagentes**

- Guanidina (6M)−EDTA (0,2 M) (pH 8,00): A solução de trabalho pode ser preparada misturando cloridrato de guanidina, grau para biologia molecular ≥99% (Ref: G3272, Sigma-Aldrich, MW: 95.53 g/mol) com 174,5 ml H_2_O grau para biologia molecular (Ref. W4502, Sigma-Aldrich) e filtrar através de um filtro de 0,22 µm.
- Glicerol (99% de pureza, Ref: G5516, Sigma-Aldrich): Autoclavado.
- Mili -QH_2_O: Autoclavado.

**Materiais:**

- Pipetas sorológicas de 5 ml.
- Tubos Falcon de 15 ml.
- Criotubos com tampa de rosca de 2 ml.
- Criotubos com tampa de rosca de 5 ml.
- Tubo tratado com 4 ml de EDTA-K2 (tampa roxa) (quantidade 2)
- Tubo não tratado de 10 ml com gel pró-coagulante (tampa vermelha) (quantidade 1),

**Protocolo:**

1. Aplique a etiqueta em todos os tubos a serem usados para processar as amostras e cubra cada etiqueta com fita plástica transparente.

Nota 1: É importante etiquetar todos os tubos antes de iniciar o processamento das amostras, para evitar misturar os tubos ao processar várias amostras ao mesmo tempo.

Nota 2: Os volumes de amostras descritos neste protocolo correspondem a amostras de pacientes com 18 anos ou mais. Embora os volumes usados no processamento de amostras de participantes abaixo dessa idade sejam diferentes, o procedimento de processamento sempre será o mesmo.

- **Sangue total (10 ml): Recolhido em dois vacutainer individuais, tubos com tampa violeta. Duas amostras diferentes são obtidas desses tubos:**
  - Sangue total + guanidina (tubo 1):

1. Transfira o volume total de sangue de um dos tubos para um tubo Falcon de 15 ml.
2. Adicione o mesmo volume de cloridrato de guanidina (6M) – EDTA (0,2M).
3. Misture por inversão e aliquote o volume resultante em dois criotubos de 5 ml com tampa de rosca.
   - Plasma (tubo 2):
4. Centrifugar a amostra do tubo tratado com EDTA com sangue total a 1200 g durante 10 minutos à temperatura ambiente.
5. Transfira o máximo volume possível do plasma para um frasco de 15 ml, sem mexer no material precipitado (*pellet*).
6. Transfira aproximadamente 1/3 do volume de plasma e prepare alíquotas de 2 ml em criotubos com tampa de rosca.
7. Adicione um volume de glicerol igual ao volume de plasma restante no tubo de 15 ml.
8. Misture por pipetagem até que a solução fique homogênea.
9. Aliquote o volume restante em criotubos com tampa de rosca de 2 ml.
10. Registre o número de alíquotas resultantes, bem como seu volume no banco de dados.
11. Armazene as alíquotas a -80 ºC.

- **Sangue total em tubo não tratado para separação do soro (tubo 3, tampa vermelha)**

1. Centrifugue o tubo a 1600g por 10 min em temperatura ambiente.

Observação: Este tubo pode coagular sem centrifugação, mas isso deve ser evitado se possível.

1. Transfira o volume máximo possível de soro para um tubo Falcon de 15 ml.
2. Transfira 1/3 do volume coletado e prepare alíquotas em criotubos de 2 ml com tampa de rosca.
3. Adicione um volume de glicerol igual ao volume de plasma restante no tubo de 15 ml.
4. Misture por pipetagem até que a solução pareça homogênea.
5. Alíquota do volume restante em criotubos com tampa de rosca de 2 ml.
6. Registre o número de alíquotas resultantes, bem como seu volume no banco de dados
7. Armazene as alíquotas a -80 ºC.

- **Sangue total em tubo tratado com heparina (ou equivalente) para ensaios LAMP (tubo 4)**

1. Prepare 2 alíquotas de 250 µl.
2. Anote o número de alíquotas resultantes, bem como seu volume no conjunto de dados e armazene as alíquotas a -80 ºC.

- **Saliva (tubo 5)**

1. Centrifugue a 1000 g por 5 minutos para separar o muco.
2. Armazene o volume máximo de amostra possível em um criotubo de 2 ml.
3. Anote o número de alíquotas resultantes, bem como seu volume no banco de dados e armazene as alíquotas a -80 ºC.

- **Urina (tubo 6)**

1. Prepare três alíquotas em criotubos de 2 ml.
2. Anote o número de alíquotas resultantes, bem como seu volume no banco de dados e armazene as alíquotas a -80 ºC.

**Resumo das amostras coletadas:**

No total, devem ser obtidas 16 alíquotas por paciente:

- 2 tubos com aproximadamente 5 ml de sangue total+guanidina. Armazenar a 4ºC.
- 2 tubos com aproximadamente 2 ml de plasma+glicerol e 1 tubo com aproximadamente 1 ml de plasma sem glicerol. Armazenar a -80ºC.
- 2 tubos com aproximadamente 2 ml de soro+glicerol e 1 tubo com aproximadamente 1 ml de soro sem glicerol. Armazenar a -80ºC.
- 2 tubos com aproximadamente 250 µl de sangue heparinizado. Armazenar a -80ºC.
- 3 tubos com aproximadamente 2 ml de saliva. Armazenar a -80ºC.
- 3 tubos com aproximadamente 2 ml de urina. Armazenar a -80ºC.

**Formato proposto para o conjunto de dados de armazenamento de amostra** ( xls )

| **CÓDIGO DA ETIQUETA** | **TIPO DE AMOSTRA** | **DATA DE PROCESSAMENTO** | **VOLUME** | **LOCALIZAÇÃO** | **RESPONSÁVEL PELO PROCESSAMENTO** | **OBSERVAÇÕES** |
| --- | --- | --- | --- | --- | --- | --- |
|  |  |  |  |  |  |  |
|  |  |  |  |  |  |  |
|  |  |  |  |  |  |  |
|  |  |  |  |  |  |  |
|  |  |  |  |  |  |  |

**Modelo de etiqueta sugerido**

| CHA 1-1111  __01/01/2022__  SUG |
| --- |
