# Supplementary material for: A guide for the generation of repositories of clinical samples for research on Chagas disease
Source: PLoS Negl Trop Dis. 2024 Aug 15;18(8):e0012166. doi: 10.1371/journal.pntd.0012166 (PMC11326570; doi:10.1371/journal.pntd.0012166)
Supplement: S1 Acknowledgements — (DOCX) [file pntd.0012166.s009.docx]

NHEPACHA Network study group: Janine Ramsey, Angelica Pech May, Alba Valdez Tah, Gilberto Sanchez Gonzalez, Adriana Gonzalez Martinez, Eduardo Ortiz Panozo, Mario J. Grijalva, Jaime A. Costales, Cesar A. Yumiseva, Carolina Herrera, Eileen Velez, Maria de Lourdes Torres, Maria-Jesus Pinazo, Sergio Sosa Estani, Colin Forsyth, Eric Chatelain, Ivan Scandale, Fabiana Barreira, Tayná Marques, Marina Certo, Alejandro Hasslocher, Roberto Saraiva, Mauro Mediano, Andrea Silvestre, Sergio Xavier, Luiz Sangenis, Fernanda Mendes, Gilberto Sperandio da Silva, Andrea Costa, Henrique Veloso, Marcelo Holanda, Flavia Mazzoli, Paula Simplício da Silva, Tania Araujo, Mariana Wagabi, Luciana Garzoni, Constança Brito, Roberto Ferreira, Rita Machado, Raquel Aguiar, Marcelo Abril, Soledad Beron, Alejandro Schijman, Silvia Longhi, Arturo Muñoz-Calderón, Belkisyole Alarcon de Noya, Oscar Noya Gonzalez, Arturo Muñoz, Cecilia Colmenares, Ivan Mendoza, Zoraida Diaz, Raiza Ruiz, Ana Andreina Alviares, María Carmen Thomas, Manuel Carlos Lopez, Adriana Egui, Celia Benitez, Inmaculada Gómez, Francisco Macias Huete, Andres Mariano Ruiz, Rocio Rivero, Mónica Esteva, Margarita Bisio, Marisa Fernandez, Yolanda Hernandez, Julio Alonso Padilla, Joaquim Gascon, Irene Losada Galván, Nieves Martinez Peinado, Juan Carlos Gabaldon, María Gabriela Alvarez, Lococo Bruno, Laucella Susana, Flavio Andrés Tóman Conte, Dr. Enrique Morral, Maria Cecilia Albareda, Fernán Agüero, Emir Salas Sarduy, Alejandro Ricci, Leonel Bracco, Mercedes Didier Garnham, Alejandro Luquetti, Igor Almeida, Ester Sabino, Felipe Guhl, Faustino Torrico.
